# Spatial attention alters visual cortical representation during target anticipation

**DOI:** 10.1101/2024.03.02.583127

**Published:** 2024-03-07

**Authors:** Ekin Tünçok, Marisa Carrasco, Jonathan Winawer

**Affiliations:** 1 Department of Psychology, New York University, New York, NY, 10003, USA; 2 Center for Neural Science, New York University, New York, NY, 10003, USA

**Keywords:** spatial attention, anticipatory response, fMRI, population receptive fields, psychophysics

## Abstract

Attention enables us to efficiently and flexibly interact with the environment by prioritizing some image features, such as location or orientation, even before stimulus onset. We investigated how covert spatial attention affects responses in human visual cortex prior to target onset and how it affects behavioral performance after target onset, using a concurrent psychophysics–fMRI experiment. Performance improved at cued locations and worsened at uncued locations, relative to distributed attention. BOLD responses in cortical visual field maps changed in two ways: First, there was a stimulus-independent baseline shift, positive in map locations near the cued location and negative elsewhere. Second, population receptive field centers shifted toward the attended location. Both effects increased in higher visual areas. Together, the results show that spatial attention has large effects on visual cortex prior to target appearance, altering neural response properties across the entirety of multiple visual field maps.

## INTRODUCTION

The visual system needs to prioritize behaviorally relevant locations because it has limited capacity to fully process all neural inputs simultaneously^1–4^. Attention is a mechanism that selectively prioritizes some aspects of information over others. In covert spatial attention, the prioritized location can be varied while keeping the fixation point constant, which enables the study of spatial properties of top-down attention in retinotopic coordinates across the visual field. Covert spatial attention improves behavioral and neural sensitivity to a stimulus at the attended location at the cost of worsening the processing at other locations^4–8^.

Two effects of covert spatial attention on neural responses have been reliably found. First, it increases response amplitude for neurons with receptive fields near the attended location, measured in both animal single unit studies and human fMRI, in striate and extrastriate areas^9–13^. Second, it modulates the preferred position and size of single neuron receptive fields (RFs)^14,15^ and population receptive fields (pRFs)^16–20^. These studies used experimental designs in which the neural measurements were made in response to the attended stimulus, or to another stimulus, such as a retinotopic mapping stimulus, while the attended stimulus was viewed.

The effects of attention begin, however, before the appearance of an attended target, once the task relevance of a particular location is established. For example, in non-human primates, V4 neurons increase their baseline activity prior to the attended target appearance^10^. In humans, attention affects the BOLD signal in visual cortex prior to target appearance^21–23^. Moreover, contralateral alpha oscillations prior to target appearance gate information flow to enhance the visual cortical representation of an attended feature or location^24–27^. These pre-target attentional effects are predictive of performance on the subsequently presented target^28–32^.

It is unknown to what degree similar circuits are involved during target anticipation, which is purely top-down, and attentional modulation of stimulus evoked responses, in which top-down and stimulus-driven effects interact. Moreover, studies of attention during stimulus presentation have led to conflicting conclusions about whether spatial attention primarily works by changing neural position tuning^19^ or response amplitude^33^. The finding in macaque V4 that distinct neuronal populations are modulated by pre- and post-stimulus attentional signals^34^ suggests that human fMRI evidence on the attentional modulation of responses to an attended stimulus may not generalize to the anticipatory effects of attention. In particular, how receptive field properties and amplitude modulations are affected by attention before stimulus onset is an open question.

Here, we investigated the spatial organization of anticipatory attentional effects in human visual cortex by implementing a concurrent fMRI/psychophysics experiment. We manipulated the location of endogenous (voluntary) attention in each trial with an event-related design. During the period between cue and target, a pRF mapping stimulus, irrelevant to the participant’s task, was briefly presented. This enabled us to measure how response amplitudes to the task-irrelevant mapping stimuli were influenced by attention for the upcoming target. Because the pattern of response amplitudes across different bar stimuli determines the pRF location, we could also assess how attention affected pRF position. Moreover, because participants did a task at the end of each trial, we were also able to measure performance benefits (valid cues) and costs (invalid cues) of attention, relative to distributed attention (neutral cues). Together the experimental results revealed robust effects of attention on behavior, and changes in BOLD amplitude and pRF position throughout the entirety of multiple visual field maps (from V1 toLO1). Together, these results provide compelling evidence that anticipatory spatial attention changes basic neural response properties throughout the cortical visual processing hierarchy.

## RESULTS

**Figure 1.**
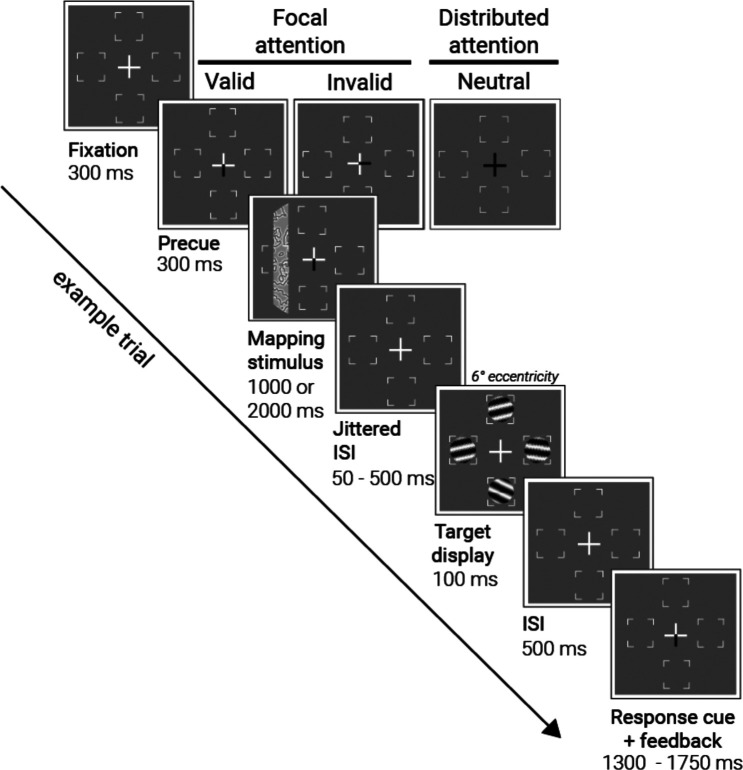
Concurrent psychophysics and pRF mapping protocol. Each trial started with a fixation cross, which then turned into an attentional pre-cue indicating one of four likely target locations (focal attention, 80% of total trials) or all four locations (distributed attention). The pre-cue was followed by one of 49 mapping stimuli as participants sustained their attention at the cued location. After a jittered ISI, the target and distractors, four Gabor patches, appeared. Then, a response cue informed participants on which stimulus’ tilt to report (clockwise vs counterclockwise). In 60% of trials, the response cue indicated the same location as the pre-cue (*valid trials)*; in 20% of trials, it indicated a different location (*invalid trials)*. The remaining 20% were neutral trials where the attentional cue was not informative. Participants received accuracy feedback by a color change in the fixation cross upon responding. The attentional cue (one of five) and the mapping stimulus (one of 49) varied from trial to trial in a pseudo-randomized design.

Participants (*n*=8) performed a two-alternative forced-choice orientation discrimination task while attention was manipulated in the scanner (Figure 1). On each trial, a central pre-cue indicated the likely target location: one of the four cardinal meridians (focal attention) or all four locations (distributed attention). Then, participants viewed a pRF mapping stimulus, followed by an array of four Gabor stimuli. A response cue at the end of each trial indicated which of the four stimuli to discriminate (CW/CCW of horizontal). For each of the five attention conditions, we measured (a) behavioral sensitivity to the Gabor target to quantify the selective attentional effect on performance and (b) the BOLD response to the mapping bar stimuli to quantify the attentional effect on response amplitude and position tuning.

### Attention improves performance at the attended location and impairs it at unattended locations

For each participant, we estimated the tilt angle threshold at each Gabor location to equate baseline performance at 82% for each target (in an experimental room). Thresholds revealed typical performance fields^35–37^; mean thresholded tilt angle across participants was 11.6° (68% CI bootstrapped: 7.4°–15.8°) and 11.4° (7.3°–15.9°) at left and right horizontal meridians, and 15.8° (9.2°–22.7°) and 21.6° (16.8°–26.1°) at lower and upper vertical meridians. Individual thresholds were used in subsequent psychophysics measurements during the scanning sessions, to equate the baseline performance across locations within and across participants.

**Figure 2.**
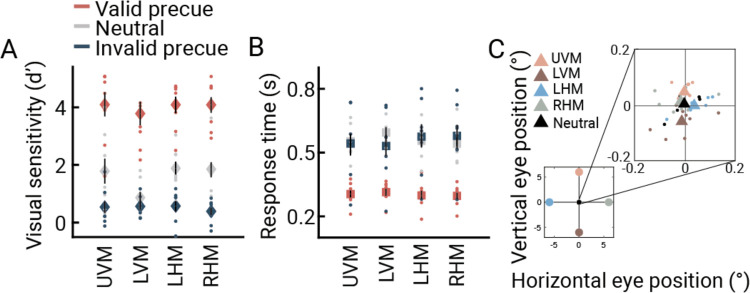
**A)** Behavioral sensitivity (d′) as a function of cue validity at four target locations (UVM: upper vertical meridian, LVM: lower vertical meridian, LHM: left horizontal meridian, RHM: right horizontal meridian). Sensitivity was higher for valid and lower for invalid than for neutral cue trials. Filled circles indicate participant values. **B)** Reaction time (s) as a function of cue validity. Valid cue trials yielded the fastest responses, ruling out speed-accuracy trade-offs. Reaction time did not differ between invalid and neutral cues. Error bars indicate the 68% confidence interval computed across bootstrapped participant means. **C)** Participant- (circles) and group-level (triangles) averaged gaze position for different pre-cue types revealed only minor deviations from fixation point (≤0.05 deg). This figure is produced by *fig2_A_B_dprime_RT.m* and *fig2_C_averageGaze.m*.

Attention had a large effect on performance. We calculated sensitivity (d′) at each target location for valid, neutral, and invalid cue types (Figure 2A). The sensitivity for validly cued trials was more than double the sensitivity for neutral pre-cue trials, indicating that attention improved performance: across participants and the four locations, the mean d′ for valid trials was 4.0 (3.7–4.4) vs 1.6 (1.3–1.8) for neutral trials. Further, sensitivity was lower for invalidly cued trials, with a mean d′ of 0.5 (0.4–0.7), indicating that attention impaired performance at the unattended locations, imposing a behavioral tradeoff. An ANOVA yielded a main effect of cue type (F(2,14)=114, p<0.0001, η_p_^2^=0.94), no main effect of location (F(3,21)=1.63, p=0.23, η_p_^2^=0.19), as expected, and no interaction between location and cue type (F(6,42)=2.26, p=0.10, η_p_^2^=0.24). Further, reaction times ruled out speed-accuracy tradeoffs (Figure 2B). Participants were about twice as fast in the valid (*M*=0.30 sec [0.28–0.32]) than neutral (*M*=0.56 sec [0.53–0.59]) or invalid (*M*=0.56 [0.50–0.61]) trials. Finally, the average gaze position was within 0.05° of the fixation point in each of the five cue types. Good fixation is essential to measure covert spatial attention and accurate pRF position during focal attention.

### pRF model fits are accurate

**Figure 3.**
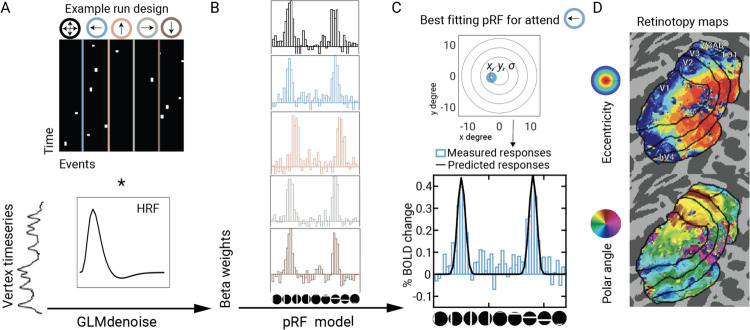
Trial-based analysis of functional time series data. **A)** In the first step, raw time series data from each vertex were submitted to a general linear model. Vertex responses were modeled as a weighted combination of the BOLD activity to distinct mapping stimulus bar locations (48+1 blank) for four different focal attention conditions and the distributed attention condition. **B)** Estimated beta weights were converted to percent BOLD change and reorganized and concatenated to create a pseudo time series for each vertex separately for each attention condition. **C)** Beta weights were submitted to the pRF model to estimate the pRF of each vertex separately for each attention condition. **D)** Vertex-level pRF estimates were aggregated on each participant’s native surface map to draw the visual field map boundaries from V1 to LO1. Retinotopy maps from an example participant (*wlsubj138*) estimated under the averaged attention condition.

To conduct the behavioral and fMRI mapping experiments concurrently, we used an event-related design with randomized mapping stimulus position across trials. This mapping sequence differs from the more common sweeping stimulus design. We first checked the accuracy of the pRF model fits. PRF models were estimated for each surface vertex in each retinotopic map separately for the five attention cues (attend-up, attend-down, attend-left, attend-right, attend-all) from the responses to the retinotopic bar stimuli that appeared before the Gabor target and distractors. First, a single GLM was conducted for each participant, providing beta weights for each bar position (48+1 blank) crossed with each cue type (5) (Figure 3). PRF models, parameterized by preferred center (x, y) and size (*σ*), were then fit to the GLM beta estimates, rather than the fMRI time series.

The pRF model provided an accurate fit to the BOLD data from each of the five attention conditions. We quantified the median variance explained by the pRF models across vertices within an ROI for each participant and each attention condition. We then averaged this value across participants, to obtain R^2^ values per attention condition per visual field map. Model accuracy was similar across the five attention conditions, with ranges of 39–40% for V1, 39–41% for V2, 36–38% for V3, 31–34% for hV4, 34–38% for LO1, and 26–29% for V3A/B. We also solved the pRF models for the data averaged across the five conditions. As expected, the variance explained by this model was higher: V1–64%, V2–64%, V3–59%, hV4–56%, V3A/B-48%, LO1–58%. The relatively high variance explained by all the pRF models is important for the subsequent analyses, which make use of the pRF parameter estimates.

### Attentional modulation of the BOLD amplitude is time-locked to the cue onset and sustained throughout the trial

**Figure 4.**
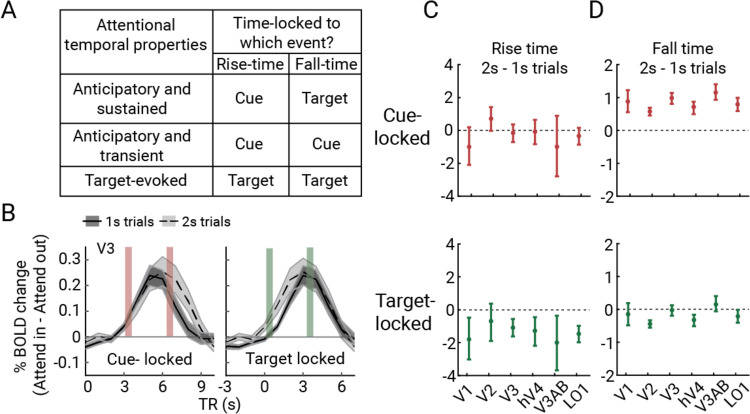
**A)** Three possibilities of the attentional modulation profile with the expected outcomes for the cue- and target-locked time series analysis. Anticipatory and sustained account predicts that participants deploy attention upon receiving the attentional cue, and sustain it until the target onset; the rise time of attentional modulation is locked to the cue onset, and the fall time is locked to the target onset. Target-evoked account predicts no anticipatory modulation, with both the rise time and the fall time locked to the attentional target onset. Finally, the anticipatory and transient account predicts that attentional modulation rises and falls prior to the target appearance; both the rise and fall time of attentional modulation is locked to the cue onset. **B)** An example of attentional modulation of BOLD in 1-s and 2-s mapping stimulus bar trials in V3, cue-locked (left), and target-locked and shifted (right). Vertical rectangles indicate the time points of interest representing the rising and falling latency of attentional modulation. **C)** Estimated latency parameters from the best-fitting logistics functions to cue-locked (top) and target-locked (bottom) BOLD activity for rising time. Latency of the rise time was around the same for 1-s and 2-s bars in cue-locked responses in contrast to target-locked responses, indicating that attentional modulation started around the cue onset. **D)** Estimated latency parameters from the best-fitting logistics functions to cue-locked (top) and target-locked (bottom) BOLD activity for falling time. Latency of the fall time was delayed in line with the prolonged anticipatory period in target-locked responses, indicating that attentional allocation was sustained until the target appearance. This figure is produced by *fig4_A_TTA_bar_duration.m* and *fig4_B_TTA_latency.m*.

An anticipatory effect predicts a sustained increase in BOLD amplitude from cue onset until target onset in cortical regions whose pRFs are close to the target. There are two other possible ways that attention might impact the BOLD response. First, an attentional modulation might start later in the trial, in response to the Gabor target, rather than to the cue. Second, an attentional response might be transient, starting after the cue but not sustaining throughout the trial. The GLM does not distinguish among the three possibilities because the Gabor target always followed the cue and the BOLD signal pools over time; hence all predict increased BOLD responses on trials when focal attention is directed toward a voxel’s pRF. Nonetheless, we can distinguish the three possibilities by analyzing the full within-trial time courses separately depending on whether the duration of the bar stimulus was one or two seconds. Though sluggish, the fMRI time series can resolve timing effects at the second or even sub-second time scale^38^. The three accounts make distinct predictions for when attention-related modulations rise and fall within a trial (Figure 4A).

First, we ask whether there is a modulation of the BOLD amplitude from focal attention. As an example, in Figure 4B we show the time course data from V3 vertices with pRFs near the cued location (cortical target ROIs; see Methods), time-locked to either the cue (top) or the Gabor target (bottom). The plots show that focal attention increased the BOLD amplitude: the time course of Attend In minus Attend Out is a positive curve, for both 1-s and 2-s trials, irrespective of how the data are time-locked. This pattern holds for all visual maps (Supplementary Figure 1).

Second, we consider the *onset* of the attentional effect on the BOLD signal. When time locked to the cue (Figure 4B, left), the attentional effect becomes apparent at about 2s, rising at the same rate for the 1-s and 2-s bar trials, consistent with the interpretation that the attentional modulation is locked to the cue rather than target. In contrast, when the time series data are aligned to the Gabor target (Figure 4B, bottom), the rise time is about a second earlier for the 2-s trials, inconsistent with the possibility that the attentional effect is time-locked to the target. To quantify these observations for each visual area, we computed the rising latency of the attentional modulation of BOLD separately for the 1- and 2-s bar trials, operationalized as the time point where the attentional modulation reached 10% of the maximum response before the peak. This point was inferred by fitting the rising portion of the attentional modulation curves with a logistic function, bootstrapped 1,000 times across participants. We then subtracted the estimated latency parameters of 1-s bar trials from 2-s bar trials (Figure 4C; Supplementary Table 1). When time-locked to the cue onset, the difference in rising latency between 1- and 2-s bar trials was close to 0 for all visual field maps (i.e., 0 was within the 68% CI of the bootstrapped estimates). Had the attentional effect been time-locked to the Gabor target rather than the cue, the difference measures would have been about +1s, inconsistent with the data in most visual field maps (all except for V2). In contrast, when time-locked to target onset, the difference was close to −1s, meaning the rise was one second earlier for the 2-s trials than 1-s trials, relative to the target. These timing differences confirm that attentional resources were allocated at an approximately fixed time following the attentional cue, rather than the Gabor target, ruling out the “target-evoked” account (Figure 4A).

Third, we considered the attentional *offset*, and whether the anticipatory response was sustained throughout the trial. Specifically, we asked whether the attentional modulation of the BOLD signal lasted a second longer when the bar stimulus lasted a second longer. We addressed this question with a similar analysis used for the onsets, but estimating the falling latency as a measure of when the attentional responses started decaying. We fit a logistic function, bootstrapped across participants, and identified the time point at which the response declined 10% from the peak. When aligned to the cue, the decline was about a second later for the 2-s trials in each visual area, indicating that attention was maintained throughout target anticipation. When aligned to the target, the latency difference was mostly zero, indicating that the decay period was aligned to the target appearance (Figure 4D), ruling out the “Anticipatory and transient” account (Figure 4A).

Together the analysis of rise time and fall time is consistent with an anticipatory account in which focal attention was initiated following the cue onset and was sustained until the target appeared, giving rise to an increased BOLD response throughout the trial. The variability in rise times is about double the variability in the fall times (error bars in Figure 4C vs 4D), suggesting more temporal variability in engaging attention following the cue than in withdrawing attention following the target. This difference is not surprising because in our protocol there were several seconds between cue and Gabor target onset; participants were not pressed to deploy attention as fast as possible.

### Focal attention affects the BOLD amplitude in a spatially tuned manner

**Figure 5.**
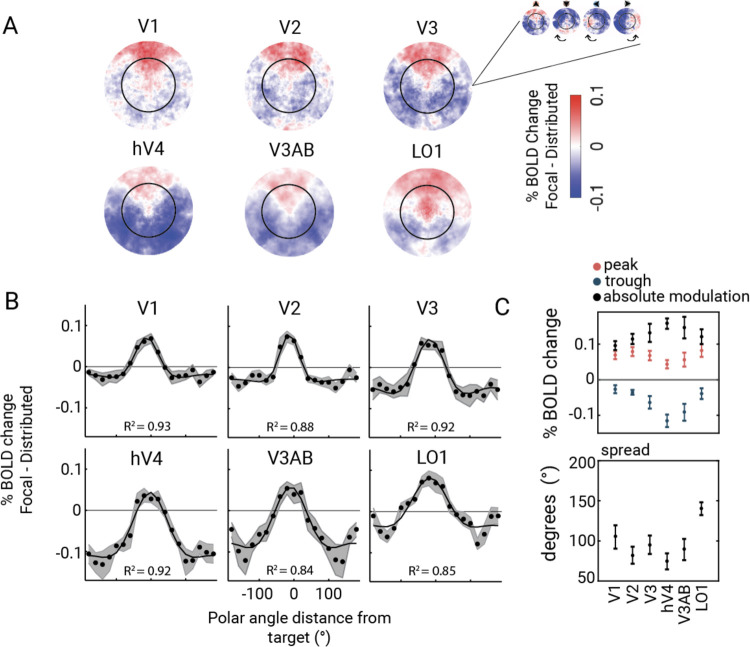
**A)** Change in BOLD response from distributed to focal attention projected onto the preferred baseline position of each vertex pRF. Attentional activity was projected onto the vertex pRF center for each polar angle target. These responses were rotated to align at the upper vertical meridian, and averaged across targets. Change in BOLD activity from distributed to focal attention was positive around the cued target location (red blobs), and negative away from the cued location (blue blobs), indicating distractor suppression fields. **B)** 2D focal attention responses collapsed onto one dimension, polar angle distance from target, representing the spatial tuning of attentional modulation around the iso-eccentric target configuration (dots indicate the measurement, solid line indicates the fits averaged across iterations) **C)** Estimated characteristics of spatial tuning of attention across visual field maps. Across maps, the peak of attentional BOLD was similar (top panel, red), but the distractor suppression magnitude varied (top panel, blue). Estimates of the attentional spread (bottom panel) showed a largely uniform spread area from V1 to V3A/B, with an increase in the spread of attention at LO1. This figure is produced by *fig5_A_2D_amplitude_plots.m* and *fig5_B_C_vonMises_fits.m*.

We next asked how the attentional modulation varied as a function of retinotopic position. Our main observation is that in all six visual field maps^a^, during the anticipatory period, there was an increase in BOLD amplitude for regions of visual cortex with pRF centers near the attended target (*target enhancement region*) and decreases for regions with pRF centers far from the target location (*distractor suppression region*). We visualize these effects in 2D stimulus space by plotting each vertex’s change in amplitude (focal cue minus distributed cue, averaged across all 49 bar positions), as estimated by the GLM (Figure 5A). The 2D space is defined by pRF centers computed from the data averaged across the 5 attention conditions. For higher SNR, we rotated the 2D maps from each of the four focal conditions to align them at the upper vertical meridian and averaged across the rotated responses and participants. A mixed ANOVA of 6 (visual field map: V1, V2, V3, hV4, V3A/B, LO1) × 2 (attention: attend in, attend out) × 4 (target location: UVM, LVM, LHM, RHM) on the averaged amplitude change of pRFs within each cortical target ROI revealed no interaction between attention condition and polar angle location (F(1,43)=2.43, *p*=0.126). The target enhancement region was a wedge shape (red) centered at the cued meridian. The shape of the target enhancement regions–narrow near the fovea and wide in the periphery–reflects increasing receptive field size as a function of eccentricity. For all six maps, the rest of the visual field (outside the wedge) tends to show decreased responses, comprising the distractor suppression field (blue). The amplitude and consistency of suppression is greater in some maps (e.g., hV4, V3A/B) than others (V1, V2).

### The pattern of enhancement and suppression varies across visual cortex

To quantify the effects of enhancement and suppression, we summarize the 2D maps as one-dimensional polar angle tuning curves, fit by a difference of two von Mises functions (Figure 5B; Table 1). A difference of two curves captures the clear center-surround organization: increased amplitude near the attended location, decreased amplitude far from the attended location. The increase in BOLD amplitude for target enhancement varied two-fold across visual field maps, ranging from 0.04 in hV4 to 0.08 BOLD% in LO1. The magnitude of suppression varied six-fold among areas, ranging from −0.02 (V1) to −0.12 (hV4) (Figure 5C). V1 and V2 showed mostly enhancement, whereas hV4 and V3A/B showed mostly suppression. The overall modulation in BOLD amplitude due to focal attention (enhancement minus suppression) generally increased along the visual hierarchy (V1<V2<V3<hV4,V3A/B). Visual areas also differed in the spatial extent of attentional enhancement, which was largest in LO1 and smallest in hV4, quantified as the degree width of the positive portion of the 1D tuning plots (Figure 5B, C). The small spatial extent in hV4 is related to its large distractor suppression magnitude (most of the tuning curve is below 0). Conversely, LO1 had the largest width and the smallest distractor suppression modulation.

### The effect of focal attention is independent of stimulus location

**Figure 6.**
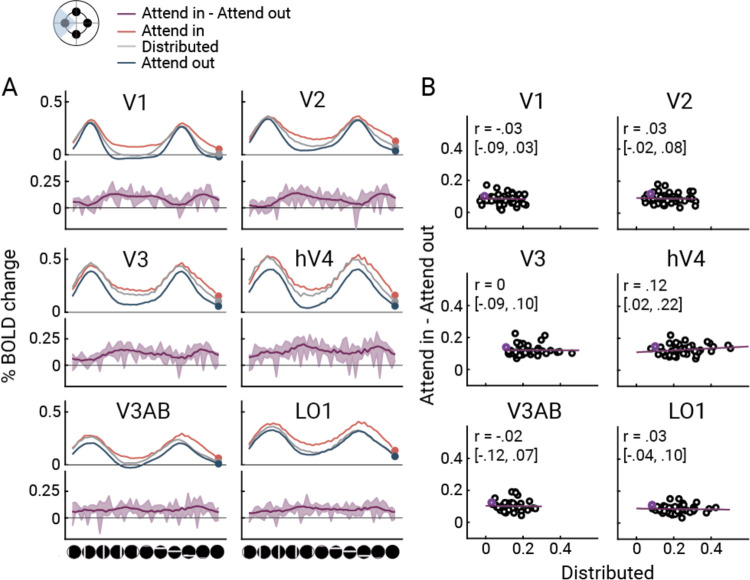
**A)** Averaged %BOLD change of left target meridian selective vertices as a function of mapping stimulus position for attend-in (red), distributed (gray), and attend-out (blue) conditions. Filled dots represent the blank presentation. Bottom section (purple) shows the difference between attend in and attend out at each bar position. Error bars of attentional modulation were computed across participant means for 68% confidence interval. **B)** Correlation between attentional modulation (attend in–attend out) and baseline response levels (distributed), averaged across polar angle locations and participants. Each dot represents a mapping stimulus position. Purple dots represent the blank presentation. Purple line represents the best fit to the group data. Correlation coefficients and 68% confidence bounds were computed by averaging and correlating the subsampled data 1000 times. This figure is produced by *fig6_A_ROI_timeseries.m* and *fig6_B_ROI_timeseries_corr.m*.

Thus far, the pre-target effects of focal attention across cortical locations show that the modulation of the fMRI signal was highly location dependent, with attentional increases in BOLD% change for vertices near the cued locations and decreases for vertices far from the cued location. Here we consider the complementary analysis, the effect of attentional modulation as a function of the mapping stimulus location rather than the vertex. To do so, we defined regions of interest, *cortical target ROIs*, comprising vertices whose pRFs are close to one of the focal targets. As an example, we plot the pseudo-time series for the cortical target ROI for the left target. The pseudo-time series consists of the response amplitudes to different bar stimuli organized by bar position, separately for distributed attention, “attend in” (focal attention to the left) and “attend out” (focal attention to one of the other three locations, averaged across the three locations). As expected, we see two peaks, one when the horizontal and one when the vertical bar stimuli overlap the receptive fields (Figure 6A). Across visual field maps and stimulus positions, the distributed attention responses tended to fall in between the attend in and attend out responses, consistent with the pattern of behavioral effects shown in Figure 2.

Across the six visual field maps, the effect of focal attention was largely independent of the mapping stimulus. First, even in the absence of a bar stimulus (“blank” condition), the BOLD amplitude differs between attend-in and attend-out (Figure 6A, dots). Second, for most visual field maps, there is a difference between attend-in and attend-out across the different stimulus locations. Third, to more directly assess whether the attentional modulation depends on the baseline response (the response amplitude to neutral cues), we plotted response to attend-in minus response to attend-out against response to the distributed cue (Figure 6B). These plots enable us to distinguish among several possible ways in which attention could modulate the responses. A positive correlation would indicate multiplicative gain, a modulation amplitude proportional to the baseline response. A flat line above the x-axis would indicate an additive shift –an increase in response amplitude for attend-in irrespective of the bar position. The data are most consistent with an additive shift: no correlation between the attentional modulation and the responses to the mapping stimuli under distributed attention. Averaged Pearson’s correlation coefficients across participants were around zero in all visual field maps, except for a very small positive correlation in hV4 (median and confidence intervals from bootstrapping in Figure 6B). We interpret this baseline shift as an anticipatory effect of attention, meaning that anticipation of the cued target increases the baseline BOLD amplitude at the appropriate retinotopic locations, independent of the mapping stimuli.

### pRF centers shift towards an attended location

We just showed that for vertices with pRF centers close to a cued location, the effect of attention was additive; an approximately fixed increment in BOLD amplitude irrespective of the bar position. There are two reasons, however, why we might expect to be able to find shifts in pRF centers. First, small differences in the effect of attention on BOLD responses to just a few bar positions near the pRF center could shift the pRF center toward an attended location, as observed previously^16,18^. Second, the conclusion about additive effects was based on analysis of only those vertices with pRFs centers close to targets; for pRFs whose centers were further away, other patterns might be observed. Here we quantified shifts in pRF centers as a function of the attentional cue.

**Figure 7.**
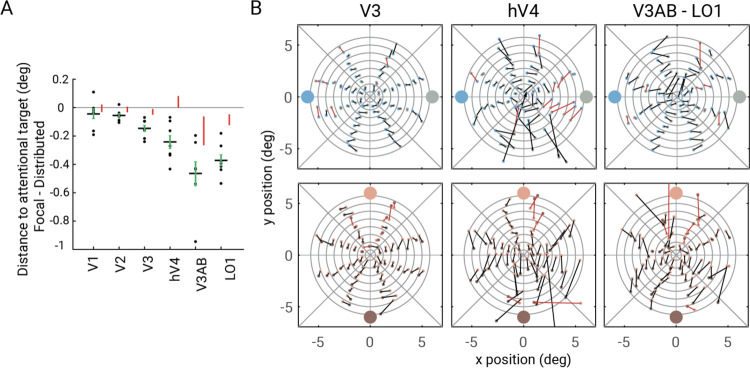
**A)** pRF centers were attracted towards an attended target. For each visual field map, averaged distance change to an attentional target from distributed to focal attention is plotted. PRF center changes were quantified by comparing the distance of the distributed and the focal attention pRF center from an attentional target. In all visual field maps, the distance to an attentional target was smaller under focal attention condition, with the effect magnitude increasing with cortical hierarchy. Green and red error bars represent the 68% confidence interval across bootstrapped participant means, the former for the correct attention condition-attended target pairing and the latter with a shuffled pairing. Dots indicate participant data. This figure is produced by *fig7_A_average_spatial_shifts.m*. **B)** PRF center shifts visualized along the horizontal (top) and vertical (bottom) attentional shift axes in V3, hV4 and V3A/B/LO1 averaged. At each bin, the vector color indicates whether the center shift occurred towards (black lines) or away (red lines) from the attentional target. Overall, pRF center shifts are in the expected direction, in line with the attentional shift. This figure is produced by *fig7_B_directional_vector_graphs_of_shifts.m*.

To estimate pRF shifts, within each visual field map, we computed the average pRF center distance to an attentional target when focal attention was deployed to that target and compared this to the distance of the pRF centers when attention was distributed. In all maps, the distance decreased during focal attention, consistent with a shift toward the attended location (Figure 7A). The decrease was slight in V1 (*M=*−0.04°, 68%CI=[−0.07,0]), and increased 10-fold along the cortical hierarchy (V3A/B: −0.46°[−0.54,−0.38]; LO1: *M=*−0.37°[−0.41,−0.33]). This indicates that anticipatory attentional signals bias the position preference of pRFs in visual cortex, and increasingly so up the cortical hierarchy. As a control analysis, we calculated the distance to a distractor 90° away from the target instead of to the cued target. The shifts tended to be close to 0, and always less than the shift to the target (Figure 7A, red bars).

We then examined the pattern of shifts as a function of pRF location in maps with the largest shifts: V3, hV4, V3A/B, LO1. To do so, we visualized the changes in pRF centers for paired cue conditions (upper vs lower, left vs right) for vertices binned within regions of the visual field (annulus sectors; Figure 7B). Overall, the pRF center shift direction was congruent with the attentional shift axis for both horizontal and vertical cue locations. The magnitude of pRF center shift increased from fovea to periphery, and increased from V3 to the higher maps. These patterns are not an artifact of binning, a problem that has affected some reports on attention-related shifts of pRFs^39^. The same patterns hold whether the binning of vertices is based on the average pRF location (attend-up and -down, or attend-left and -right) or based on independent data, i.e., pRFs solved from the neutral cueing (Supplementary Figure 2). Moreover, the results shown in Figure 7A are not binned.

## DISCUSSION

We investigated the cortical specificity, stimulus specificity, and spatial tuning of anticipatory attentional signals in human visual cortex by implementing an event-related pRF mapping protocol that enabled simultaneous measurements of BOLD and behavior. Perceptual sensitivity increased at the attended location and decreased elsewhere. Visual cortical responses across the entirety of visual field representation changed in two main ways under focal attention in anticipation of the target. First, in parallel with behavioral benefits and costs of attention, the response amplitude of vertices whose pRFs were near the target location increased and far from the target location decreased. While the magnitude of target enhancement was mostly similar across visual field maps, that of suppression varied. Second, attentional modulation of response amplitude was approximately uniform as a function of mapping stimulus position, indicating a baseline shift in responses rather than a change in response gain. Third, the pRF preferred centers were shifted towards the anticipated attentional target in all visual field maps, and increasingly so up the cortical hierarchy.

### Importance of the neutral condition in an attentional experiment

A novel feature of our experiment was measuring pRFs while manipulating the attended location on a trial by trial basis. A disadvantage of the event-related design is that it is less efficient for pRF mapping^40,^ but we compensated for this by increasing the number of scans. The trial-by-trial attention manipulation differs from previous fMRI studies on pRFs and attention, which used either designs in which the cue was always valid and the task was always performed at the cued location; the attended location was constant within a scan of several minutes^17,19^ or attention continuously tracked the mapping stimuli^16,18^. A benefit of our design is that it enabled us to measure the performance differences among valid, neutral, and invalid cueing. A hallmark of spatial attention is that it imposes tradeoffs: performance improves at the attended location and worsens at unattended locations^3,5,41–43^. Our trial-by-trial design enabled verification of the attentional task in our fMRI study. The selective nature of attention was confirmed by our behavioral results, which showed large differences among the three conditions, valid better than neutral (benefit) and invalid worse than neutral (cost). This provided a secure footing for examining the positive and negative neural effects of attention on neural responses.

The selective nature was assessed neurally on pre-target BOLD by comparing the effect of focal vs distributed cues and by comparing responses at the attended and unattended locations. When assessing the benefits and costs of attention, it is important to consider what the neutral reference point is. In psychophysical protocols, it is common to quantify attentional benefits and costs against a distributed attention baseline condition^6, 41, 43, 44^. In contrast, in electrophysiology, the comparison is often between “attend-in” and “attend-out,” which both entail focal attention^1,8,45^. Without a neutral condition, there is ambiguity about whether response differences between the two conditions arise from a change at the attended location (enhanced responses), at the unattended location (suppression), or a combination. Similarly, fMRI mapping experiments on spatial attention tend to either compare the effects of attending two locations^13,17,19,20^, or use attend-fixation as baseline^16,19,46^. Attending fixation or attending a focal location in the periphery is likely to result in withdrawal of resources from the rest of the visual field, making it difficult to assess local target enhancement and distractor suppression effects. Using distributed attention as a baseline removes these potential ambiguities. We illustrate how the choice of the neutral condition can affect estimates of both attention-related enhancement and suppression (Supplementary Figure 3). Consistent with this schematic, our results showed that the BOLD response amplitude to the mapping stimulus bars under distributed attention primarily falls in between *attend-in* and *attend-out* states, confirming that it constitutes an effective baseline measurement.

### Spatial attention induces extensive pre-target changes throughout the representation of the visual field

Anticipation enables people to successfully interact with the environment. Anticipation plays an important role in many cognitive phenomena like working memory^47^ and expectation from stimulus probability^48,49^. Attention directs anticipation as to when, where or which information in the environment needs to be prioritized and further processed than the rest, guiding action production and memory^50,51^. Thus understanding how anticipatory responses in attention affect the nervous system is an important goal in cognitive neuroscience. Doing so requires separating the effects of anticipation from the effects of the onset of the attended target. The most straightforward way to do so is to measure neural responses in the absence of a target by having long delays between cue and target. And indeed, several fMRI studies have characterized anticipatory modulation of BOLD response amplitude in the absence of visual stimulation^21–23,28–30^. Such studies consistently reveal changes in BOLD amplitude during anticipation. These studies cannot, however, measure either the effect of attention on position tuning, which requires presentation of a stimulus at a variety of target locations.

Our design allowed us to characterize three systematic effects simultaneously. First, the modulations in signal amplitude affected the entirety of visual field maps in each map studied (increases near the cued location, decreases elsewhere, i.e. push-pull dynamics). Second, in regions near the cued location, response increases were largely non-specific to the mapping location, meaning that the attentional modulation was not limited to just mapping stimuli that were close to the cued location. Third, pRF preferred positions shifted toward the focally attended target. This effect was seen throughout each of the visual maps, with overall larger shifts in later visual field maps. Together, these systematic patterns imply that attentional anticipation reshapes responses throughout the visual maps.

### A multiplicity of effects of attention on BOLD amplitude

Single neuron studies show that spatial attention can induce both additive^52, 53^ and multiplicative^54–57^ effects. The simplest multiplicative effect is a scaling of the response amplitude; however, more complicated patterns can emerge from gain changes if the gain modulation acts on the inputs. For example, a shift in the contrast response function or in receptive field location have each been interpreted as changes in *input* gain. For position shifts, this manifests as a multiplication of an attentional gain field with a neural receptive field^15, 45^. If the attention field and the receptive field differ in center location or in size or both, then input gain causes a shift in the receptive field, rather than a response gain.

**Fig 8.**
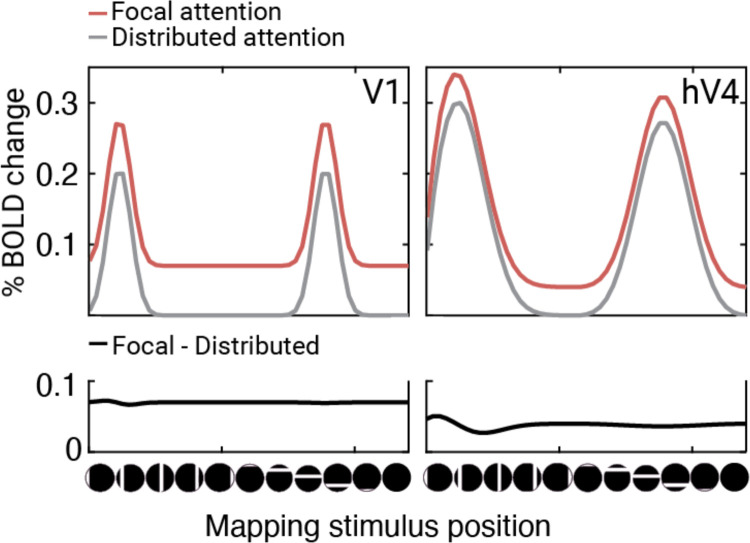
A simulation of the BOLD response to the 49 mapping stimuli in V1 and hV4. The upper panel shows separate traces for focal and distributed attention. The simulations assumed typical baseline shifts from our datasets (V1:0.07%, hV4:0.04%) and typical position shifts (V1:0.04°, hV4:0.3°). The bottom panel plots the difference between focal and distributed attention, showing two effects: a mean above 0, indicating the baseline shift, and a modulation around the mean caused by the position shift. This figure is produced by *fig8_simulateShifts.m*.

We found clear evidence for two of these three effects: a baseline shift and a position shift. As in the single unit data^15^ and computational models^15,45^, we interpret the position shift as a gain modulation on inputs. The shift effect is, by definition, stimulus dependent: the pRF center location depends on which bar positions elicit the largest responses. We interpret the baseline shift to indicate that anticipation of the target increased neural activity independent of the mapping stimulus. This interpretation is supported by the observation that focal attention increases the BOLD response during blank trials, when no mapping stimulus is present. The two effects are measured in different units –the baseline change is measured in response amplitude (percent BOLD) and the shift is measured in degrees of visual angle. We can, however, make a comparison by estimating how much each effect influences the response amplitude as a function of bar position (Figure 8). This shows that the baseline shift is the dominant effect in V1, whereas both the position shift and the baseline shift are substantial in hV4. Note that the effect of position shifts on the pattern of responses to the bars is not evident when averaging across a region of interest (Figure 8) because the direction of the shift differs across voxels.

We did not observe a change in response gain. The most likely explanation lies in the type of attention we studied. We deliberately isolated anticipatory responses from the stimulus-evoked modulation. The task-relevant target (the Gabor) was low contrast, small and briefly presented to minimize its effect on the BOLD. The mapping stimulus was designed to dominate the BOLD signal but was not relevant to the task. It is likely that, had the mapping stimulus been task-relevant, we would have observed a gain change. Indeed, attention-related changes in response gain have been observed in an experiment in which participants performed a task on the mapping stimulus ^(18)^. A less likely explanation is the possibility that fMRI is generally insensitive to the type of gain modulations caused by attention. This might seem plausible because several fMRI studies measuring the effect of spatial attention on the contrast response function found a baseline shift rather than a gain change^46,58–61^, in contrast to electrophysiology studies which report both^52–55^. Some have speculated that the discrepancy may be due to an inherent insensitivity of the BOLD signal to gain modulation^62, 63^. We think the limitation is more likely due to the complexities of how gain changes affect the pooled response of a diverse neural population^64^. This is supported by two observations. First, a recent fMRI study on feature-based attention observed a gain change in the contrast response function^65^. Second, as noted above, several studies on spatial encoding (rather than spatial contrast) have reported robust attention-related gain effects, either in terms of response gain^18^, or position shifts^17, 19^. Hence it seems likely that were there a large change in response gain, our protocol could have detected it. Rather, anticipatory spatial attention seems to have large effects on baseline response and input gain, with less effect on response gain.

### Attentional enhancement and suppression may recruit cortical areas differentially

The amplitude of attentional modulations, particularly in terms of distractor suppression, varied across cortical areas. Suppression magnitude increased along the cortical hierarchy from V1 to hV4. The pattern of target enhancement was quite different. In fact, the map with the largest suppression effect, hV4, had the least enhancement and conversely, the map with the largest enhancement, LO1, had little suppression. If the two effects were mediated by the same mechanism, we would expect that enhancement and suppression would covary across maps, contrary to our results. Our results are more consistent with the idea that there are at least partially independent circuits mediating target enhancement and distractor suppression in visual cortical areas, as suggested by some psychophysical and neural results^66–69^.

The spatial extent of the attentional effects was relatively similar across maps. With the exception of LO-1, there was a sharp transition from enhancement to suppression, with about one quarterfield of enhancement and three quarterfields of suppression. The sharp transition indicates that the top-down signals mediating anticipatory target selection are represented with high precision in visual cortex. The similar spatial extent across most maps is compatible with a single attentional field modulating responses in multiple maps inferred by a model-based analysis of how attention alters pRF location^17^. We confirm it with a direct measure of the spatial extent of amplitude modulation.

#### Polar angle asymmetries.

We chose four target locations on the four cardinal meridians because visual performance is known to differ at these locations. Specifically, performance is typically best along the horizontal, intermediate along the lower vertical, and poorest along the upper vertical meridian^70^. Certain structural and functional differences in the retina^71,72^ and cortex^73–76^ might underlie these asymmetries. We equated discriminability in a pre-scan experiment without focal attention by choosing orientation deviations that yielded the same performance at all locations, which showed the largest tilt angle on the upper vertical meridian, and smallest on the horizontal. Once equated, performance for each neutral, valid and invalid trials did not differ across meridians during the scanning experiment, consistent with the finding that endogenous attention improves performance similarly at iso-eccentric locations^77^. In addition to replicating this behavioral finding, we also show neural evidence that attentional modulations are similar at different iso-eccentric locations, when baseline performance is equated.

## CONCLUSIONS

Attention alters many properties of visual cortical responses across the representation of the visual field in anticipation of a target, prioritizing the processing of the target to facilitate the subsequent stimulus-evoked activity. These effects include shifts in pRFs toward the attended location and stimulus-independent amplitude modulations that are positive near the cued location and negative away from it. The positive and negative amplitude modulations parallel the positive and negative effects on performance for valid and invalid trials, respectively. The combination of a baseline shift in BOLD amplitude and a position shift in pRFs indicate two processes by which anticipation affects activity in visual cortex: a change in input gain underlying the position shift, and a sustained change in neural activation. An important remaining question is how each of these two effects, measured in anticipation of a stimulus, alter responses to the subsequent stimulus, measured both behaviorally and neurally.

## MATERIALS AND METHOD

### Participants

Nine human participants (*M*_*age*_ = 28.5, seven females, two males) with normal or corrected-to-normal vision participated in the experiment. Following behavioral thresholding and training sessions outside the MRI scanner (about 1.5 hours), participants took part in four scan sessions (*n*=7), except for one participant who became unavailable after 3 sessions, and one participant who was excluded from the study after 3 sessions due to poor quality retinotopic maps (After participant exclusion, *M*_*age*_ = 29.1, with seven females, one male). Participants were compensated $30/h for participation. They signed a consent form approved by the New York University Institutional Review Board, and an MRI screening form prior to each scan session.

### Apparatus

For both data collected inside and outside the scanner, stimuli were generated on an Apple iMac using MATLAB (R2019) and Psychtoolbox-3^78^. Both inside and outside the scanner, the gaze position of the participants’ dominant eye was tracked at a sampling rate of 1 kHz using an Eyelink 1000 (SR Research, Ottawa, Ontario, Canada). For the behavioral thresholding and training sessions outside the MRI scanner, stimuli were displayed on a cathode-ray tube monitor with a flat screen (1280 × 960 pixels, 40.5 × 30 cm; 60 Hz). A Konica Minolta LS-100 photometer was used to linearize the display luminance prior to data collection. Participants sat 57 cm away from the monitor. Behavioral responses were collected using a computer keyboard. In the MRI scanner, stimuli were displayed on a ProPixx DLP LED projector in the MRI scanner with a linear gamma table (VPixx Technologies Inc., Saint-Bruno-de- Montarville, QC, Canada). The projected screen (60 cm × 36.2 cm) had a resolution of 1920 × 1080 with a refresh rate of 60Hz. The participants viewed the screen through an angled mirror mounted on the head coil. The total viewing distance (eye to mirror plus mirror to screen) was 86.5 cm. Behavioral responses were collected using two assigned keys of a 4-button fiber optic response box (Current Designs). Participants were placed inside the scanner with positioner pads to reduce head motion.

### Stimuli for attentional task

All stimuli were presented on a gray background (~60 cd/m^2^). A white (~120 cd/m^2^) central cross subtending 0.35° in visual angle was used for stabilizing gaze position. One or all four arms of the cross changed from white to black (~0 cd/m^2^) to cue participants to attend to one of four locations, or to distribute attention to all locations, respectively. There were four target stimuli presented at 6° of eccentricity on the four cardinal meridians (0°, 90°, 180° and 270°). Each target was a tilted Gabor patch, constructed by windowing a sine wave grating (4 cycles per degree, 10% Michelson contrast) by a Gaussian (1° full-width-at-half-maximum), truncated within a square aperture at 3° per side. The Gabor patches were tilted slightly clockwise or counterclockwise from horizontal. To eliminate position uncertainty, white placeholders (~120 cd/m^2^), positioned just outside the corners of the four apertures, indicated the target positions throughout the experiment.

### Stimuli for pRF mapping

Between the attentional cue and target, a retinotopic mapping stimulus was viewed for 1 or 2 seconds. The mapping stimulus was a contrast pattern windowed within a bar aperture (3° in width). All stimuli were limited to a circular window 12.4° in radius. The bar aperture was presented either horizontally or vertically, at one of 24 positions per orientation. The 24 vertical and the 24 horizontal bar apertures gridded the circular window in steps of 1° between two adjacent locations, with an overlap of 2°. The contrast pattern within the bars was composed of curved gray-scale contours (peak spatial frequency of 3 cpd) with Michelson contrast of 20%. These textures were found to be effective in eliciting BOLD responses across multiple retinotopic maps^79,80^.

### Task

#### Trial sequence and attentional cueing.

Each trial began with the presentation of a white central fixation cross for 300 ms (Figure 1). The cross then changed to a pre-cue, which was shown alone on the screen for 300 ms: On 80% of trials, the pre-cue consisted of one of the four arms of the fixation turning black to cue *one* of the four target locations (focal attention; equal probability at each location). On the other 20% of trials, the pre-cue consisted of all four arms changing to black to cue *all* target locations (distributed attention). In the focal condition, cue validity was 75% (i.e. a response cue matched the location of the pre-cue for the 75% valid trials, but not for the 25% invalid trials; see below). Because the task was difficult (thresholded in pre-scan testing) and the cue was informative, it was in the participant’s best interest to attend to the cued location for better performance in the task.

Following the 300 ms white cross and the 300ms pre-cue, a mapping stimulus was presented for 1000 or 2000 ms. The two durations were used to reduce expectations of when the stimulus would disappear, and the target would appear. The bar aperture did not move during this 1- or 2-s period, but the contrast pattern inside it updated to a new random sample of the texture every 250 ms. In 10% of trials, the mapping stimulus was blank to estimate the baseline BOLD response in the absence of mapping stimuli, consistent with prior pRF mapping protocols^81^. The pre-cue remained on the screen during the 1s or 2-s bar stimulus. The prolonged presentation of the pre-cue was to ensure that attention would be deployed and sustained while the mapping stimulus was displayed. The mapping stimulus was followed by a jittered interstimulus interval (ISI) of 50 ms (10% of trials), or 300, 400, or 500 ms (equiprobable at 30%). During the ISI, only the white fixation cross was presented. The two durations of the mapping stimulus and the jittered ISI imposed temporal uncertainty about the target appearance, to promote sustained attentional deployment.

The jittered ISI was followed by the four Gabor targets, which were presented for 100 ms and tilted around a horizontal reference angle in CW or CCW directions. The tilt amount was titrated to 82% baseline performance for each participant at each target location prior to scanning (see Thresholding session for details). Target display was followed by another ISI of 500 ms. Finally, a response cue, a central cross with one black arm, informed the participant of which target to make the 2AFC orientation judgment on. The participant reported the direction of the tilt by making a keyboard press.

Participants had about 1500 ms to make a response, and then received feedback. The exact maximum response window varied from 1350–1700 ms to ensure that the trial ended synchronously with the MRI volume acquisition. This trial to trial variation had no effect on performance, as responses were much faster than the maximum window (typically about 300 to 600 ms after the response cue). Participants received feedback through a color change in fixation cross from white to green for correct answers, to red for incorrect answers, and to yellow for late answers that were not registered (<1% of trials across participants).

#### Psychophysical protocols.

Participants completed four experimental protocols, three prior to scanning and one during scanning: (1) familiarization with task, (2) thresholding, (3) attentional cueing (outside the scanner), and (4) attentional cueing (during scanning). All four protocols used the same trial structure, described above. During all protocols, participants were instructed to emphasize accuracy over speed.

1. *Familiarization*. The familiarization protocol consisted of 30 trials, which were the same as those in the main experiment except that the Gabor targets were at 100% contrast and the orientation difference was large (±15° from vertical). The purpose was to ensure that participants understood the task. This was verbally confirmed before advancing to the second protocol.
2. *Thresholding*. The thresholding protocol was conducted with *neutral cues only* to estimate the tilt deviation from the reference angle needed to reach threshold performance at each of the four locations. Threshold was defined as the tilt deviation corresponding to 82% accuracy on a fitted Weibull function. The threshold was found using the best PEST adaptive procedure of “3-down-1-up”, interleaved at each target location, where the tilt angle got smaller after three consecutive accurate answers and got larger after one inaccurate answer (palamedestoolbox.org) ^(82)^. Because the thresholding experiment only contained neutral trials, the target location was identified by the response cue. At the three other locations, the tilt angle was determined by the current staircase value for the location, and the angular deviation (clockwise or counterclockwise) was random. The thresholding session incorporated one additional type of feedback informing the participants if their gaze deviation was ≥2° from the fixation cross by a change in the color of the fixation cross from white to blue. The thresholding comprised about 65 trials at each target location (~260 trials) and took about 30 min.
3. *Attentional cueing outside the scanner*. After the threshold measurements, and prior to moving to the scanner, participants completed one full session of the main experiment (~1hr, 520 trials, including all 5 cueing conditions) at the tilt angles measured in the thresholding experiment. This served as additional training for the participants, as well as assessment of the protocol to verify that the cueing protocol affected performance as expected (improved performance at the cued locations). This benefit ensured that participants were in fact using the pre-cue.
4. *Attentional cueing inside the scanner*. Upon the completion of the three protocols outside the scanner, participants took part in multiple scan sessions, performing the 2AFC orientation discrimination task described above. During each scan session, there were 10 functional scans in which participants performed the psychophysical task. Each of these scans had 52 trials and lasted 4 min 14 s; with a total of 520 trials per scanning session. Seven participants completed 2080 trials in total (across 4 scan sessions), and two participants completed 1560 trials (3 scan sessions).

### MRI acquisition

Functional and anatomical MRI data were collected on a 3T Siemens MAGNETOM Prisma MRI scanner (Siemens Medical Solutions, Erlangen, Germany) located at the Center for Brain Imaging at NYU using a Siemens 64-channel head/neck coil. Before the functional echo-planar images (EPIs) were obtained, two distortion scans were conducted in anterior-to-posterior (AP) and posterior-to-anterior (PA) phase encoding directions to correct for distortions in functional EPI scans due to susceptibility. After the distortion scans, in each scan session, ten scans of functional EPIs were obtained using the CMRR multiband sequence with a multiband factor of 6^83–85^. Each scan had 244 volumes (TR = 1000 ms, TE = 37.6 ms, voxel size: 2mm^3^ isotropic, flip angle 68°)^83,85^. For each participant, T1-weighted magnetization-prepared rapid gradient echo images (MPRAGE) (TR = 2400 ms, TE = 2.2 ms, voxel size: 0.8mm^3^ isotropic, flip angle 8°) were previously acquired for previous studies, and were reused in the current study.

### MRI preprocessing

All DICOMS were anonymized and defaced using *pydeface* (https://github.com/poldracklab/pydeface) prior to transfer from the acquisition computer to the researcher storage space. The DICOMS were then converted to NIFTIs and organized according to the Brain Imaging Data Structure (BIDS) convention^86^ using *heudiconv* (https://github.com/nipy/heudiconv). The Anatomical and functional data were then preprocessed using fMRIPrep v.20.2.1^87^. T1-weighted (T1w) anatomical images were corrected for intensity non-uniformity and skull-stripped. Brain tissue was segmented into cerebrospinal fluid (CSF), white-matter (WM) and gray matter (GM) using *fast* based on both T1w and T2w input images. Segmented images were reconstructed using *recon-all*^88^. For the preprocessing of the functional data, a reference volume and its skull-stripped version were generated and corrected for susceptibility distortions using the two distortion maps collected with opposing phase encoding directions (AP and PA). Corrected functional reference images were co-registered to the T1w anatomical reference using *bbregister*^89^. Head-motion parameters with respect to the corrected BOLD reference were estimated and the functional images were slice-time corrected using *3dTshift*^90^. Slice-time corrected functional data was resampled to the anatomical space using one-shot interpolations (incorporating transformation matrices, distortion maps and the coregistration). Preprocessed BOLD data was then resampled onto each participant’s reconstructed cortical surface using *fsnative*, and all further analyses were performed on the *fsnative* space, using the surface-based fMRI data.

### Behavioral sensitivity analysis

Each participant’s behavioral data were concatenated across all scan sessions. The effect of attention on behavioral sensitivity was calculated across four target locations as a function of attentional pre-cue validity. For each target location and pre-cue type, d′ sensitivity index was calculated using *z(hit rate) - z(false alarm rate)*. To map our 2AFC procedure into a common signal detection framework, we arbitrarily assigned one direction (clockwise) as ‘target present’ and the other direction as ‘target absent’^43,44,91^. To correct for extreme values, prior to computing d′, we added 0.5 to the number of hits and false alarms^92, 93^. Although we emphasized accuracy over speed to the participants in the instructions, we measured reaction time to assess speed-accuracy tradeoffs, specifically, the possibility that conditions with higher accuracy had slower reaction times. To calculate the 68% confidence interval, participant-level sensitivity and reaction time values at each cue type and target location were computed 1000 times by resampling the data with replacement (bootstrapping). With two-way repeated measures ANOVAs with location (UVM × LVM × RHM × LHM) and cue type (Valid × Neutral × Invalid) factors we assessed the differences among cue types both for sensitivity and reaction time.

### Gaze position analysis

Because we were interested in the effect of endogenous covert attention (a top-down process), it was critical that the stimuli were matched across retinotopic coordinates, and that participants maintained central fixation. Outside the scanner, trials began once participants were fixating for 500 ms, and trials were aborted and repeated when fixation was broken (criterion of 2°). During scanning, trials were scheduled to match the fMRI acquisition, and hence were never aborted or delayed. However, fixation breaks were rare (2% of sampled frames across participants, 68% CI = [1% – 4%]). We also analyzed the gaze position during scanning to determine whether there were systematic biases in gaze direction across different attention conditions (the five cue types). Gaze position was recorded at 1,000 Hz in X, Y screen pixels. We converted the gaze position data from *X* and *Y* positions in screen pixels to degrees in visual angle. Time points with blinks were identified by the EyeLink software and removed from analysis. For every trial, we then averaged the X, Y position across time points from the trial onset to target display onset (1.6 or 2.6 s, depending on the duration of the mapping stimulus). These time-averaged values were then averaged across all trials with the same pre-cue, yielding 5 *X*, *Y* pairs per participant. Finally, to correct for calibration errors, we demeaned the values for each participant.

### fMRI Data Analysis

Functional time series data of each vertex were analyzed in two steps^80^: First, a general linear model (GLM) was solved on the concatenated trial responses across multiple scan sessions. Second, a pRF model was fit to the estimated GLM weights. All analyses were performed on the NYU High Performance Computer cluster using MATLAB (Version 9.14.0, R2023a).

#### General linear model.

Functional time series of each vertex on each participant’s native surface were modeled with a GLM. Preprocessed functional time series were concatenated across multiple scan sessions and modeled using *GLMdenoise* (https://github.com/cvnlab/GLMdenoise)^94^. GLMdenoise is a powerful analysis toolbox that *1)* identifies and regresses out task-irrelevant signals in time series data, *2)* optimizes a hemodynamic response function for each participant, and *3)* estimates GLM weights with bootstrapping to obtain confidence intervals and cross-validation to quantify model accuracy.

A design matrix was constructed for each run with 250 predictors (columns). Of these, 245 columns were for the mapping stimuli: 5 attention conditions × 49 mapping stimuli (48 bar locations + 1 blank). For these columns, there was a ‘1’ for each TR when the mapping stimulus was present (either 1 TR or 2 TRs, because the mapping stimuli were either 1 or 2 s). There were 5 additional columns for the Gabor targets, one for each of the 5 attention conditions. These were modeled as a ‘1’ during the TR that the target appeared. The predictors did not take into account cue validity because the validity only becomes apparent after the mapping stimulus and Gabor target are viewed.

(Eq 1) $${\hat{y}}_{i}=\left(b_{M_{1}}x_{M_{1}}+\cdots b_{M_{245}}x_{M_{245}}+b_{T_{1}}x_{T_{1}}+\cdots b_{T_{5}}x_{T_{5}}\right)*HRF+N$$

where *y*_*i*_ represents the predicted time series of voxel *i*, the *b* terms are the coefficients (‘beta weights’), and the *x* terms are indicator variables (either 0 or 1). The subscripts denote the condition, with *M*_1_ to *M*_245_ corresponding to the mapping stimulus, and *T* corresponding to the Gabor targets and *N* represents the nuisance variables (polynomial regressors for detrending low frequency fMRI drift, and task-irrelevant time series regressors identified by GLMdenoise). All variables other than the nuisance regressors were convolved with a hemodynamic response function (*HRF)* estimated for each participant (a 50-s finite impulse response function). Estimated coefficients from the GLM were converted to percent BOLD change by dividing them by the mean signal intensity. For visualization purposes, estimated coefficients from the GLM representing the responses to mapping stimulus bars were reorganized as a function of mapping stimulus position, and convolved the measured responses with a triangular function that was padded with the first and the last values of the data, and then normalized by the total sum^95^.

#### pRF models.

We solved separate pRF models for each of the five attention conditions, using the 49 mapping stimulus coefficients for that condition. We also solved a pRF model for the average response across the five attention conditions by averaging the five coefficients for each of the 49 mapping stimuli. This resulted in six different preferred position and size estimates per vertex: one for each of the five attention conditions (4 focal and 1 distributed) and one for the average across attention conditions. For visualization purposes, the 49 coefficients per attention condition were ordered into horizontal and vertical sweeps (plus a blank), so that the estimated BOLD response and model predictions could be viewed as pseudo time series.

PRF analysis was conducted using *vistasoft* software (https://vistalab.stanford.edu/software, Vista Lab, Stanford University), with a custom wrapper function (https://github.com/WinawerLab/prfVista, New York University). Each vertex’s pRF was modeled as a circular 2D Gaussian, parameterized by its center (*x, y* in deg) and size (*σ*, one standard deviation of the Gaussian, in deg)^81^. The pRF model predicts the response amplitude to each mapping stimulus by pointwise multiplication of the Gaussian pRF and the binarized contrast aperture, scaled by a nuisance factor (the vertex-specific gain). Unlike many implementations of pRF models, our pRF model did not explicitly account for the hemodynamic response, low frequency detrending, or conversion to percent BOLD change, because these steps were already computed by the GLM.

The pRF fitting software estimates the pRF parameters (x, y, *σ*) by minimizing the residual sum of squared between the measured BOLD pseudo-time series (the 49 coefficients from the GLM) and the predictions from the model. We implemented a coarse-to-fine search for pRF fits for both computational efficiency and to reduce the chance of model solutions that correspond to local rather than global minima. In this procedure, the results from an initial brute force grid search were used as the starting point for a finer second stage fitting procedure^74^. The coefficient of determination, *R*_*2*_, was calculated as 1 - (RSS / TSS), where *RSS* is the sum of squares of residuals and *TSS* is the total sum of squares (i.e., the sum of squares of the data).

#### ROI definitions.

We visualized retinotopic data by projecting each vertex’s preferred position in polar coordinates onto flattened maps of the visual cortex, centered at the occipital pole, using *Neuropythy v0.12.11* (https://github.com/noahbenson/neuropythy)^96^. Boundaries of V1, V2, V3, hV4, V3A/B and LO1 were drawn manually on each participant’s native MRI surface, guided by the eccentricity, polar angle, and cortical curvature maps. The criteria for identifying map boundaries follow prior work: V1–V3^97^, hV4^98^; V3A/B and LO1^99^. We defined V3A and V3B as a single ROI because the boundary separating them was not clear in every participant.

#### Bootstrapping procedure.

We used bootstrapping procedures throughout this paper to estimate confidence intervals and to test for the statistical significance of most of our results^100^. We sampled eight participants with replacement 1000 times and calculated the 68% confidence interval based on the bootstrapped population distribution. The bootstrapped distribution was compared against a null distribution to test for statistical significance.

#### Latency analysis of attentional modulation of BOLD.

For each visual field map in each participant, we estimated the latency of attentional modulation, meaning the time during a trial at which the BOLD response differed as a function of attentional condition. To estimate the latency, we first extracted the BOLD time series of cortical ROIs representing the target locations. We converted the time series of each vertex to percent BOLD change, and detrended the converted data by projecting out a first order polynomial within each scan. We then extracted a 10-s event-triggered time series for each trial, aligned either to the attentional cue (“cue-locked”) or to the Gabor target onset (“target locked”). These event-triggered time series were averaged across trials in which the mapping stimulus bar overlapped the target corresponding to that cortical target ROI. The time series were then also averaged across vertices within the target ROI. These averages were computed separately for trials with 1-s bar stimuli and 2-s bar stimuli, and for trials in which focal attention was directed to the target corresponding to the ROI (“attend in”) and trials in which focal attention was directed to one of the other targets (“attend out”). Next, we subtracted the average BOLD time series for attend-out from attend-in trials to quantify the attentional modulation over time.

To quantify the latency of attentional modulation, we used the latency analysis method previously described^101^. We first normalized the event-triggered responses such that the minimum attentional response is equal to 0 and the maximum is equal to 1. We next extracted the time point (TRs) at which the response peaked and then separated the response curves as before and after the peak point, representing the rising and falling of attentional responses. We fit logistic functions to the two portions of the attentional response, separately to the 1-s and 2-s mapping stimulus trials of cue- and target-locked responses, using the following equation:

(Eq 2) $$f\left(t;m,t_{50}\right)=1/\left(1+\text{exp}\left(-m*\left(t-t_{50}\right)\right)\right)$$

where *t* represents time in seconds (independent variable), *m* represents the slope and t50 represents the mean. On the best-fitting functions, we defined the latency of the attentional response with a t10 parameter, the time point at which the attentional modulation reaches the 10% of the maximum response. We lastly subtracted the estimated latency of the rising time of attentional modulation in 2-s mapping stimulus trials from 1-s mapping stimulus trials. The fitting procedure was repeated 1,000 times on the group-level responses averaged across sub-samples of participant-level responses with replacement.

#### 2D visualizations of attentional activity.

To investigate the effects of focal attention on BOLD amplitude, we constructed visualizations of attention effects in visual space. We did this by first constructing separate visual field representations of each visual area for each of the four focal attention conditions for each participant. For a given participant and visual field map, we assigned each vertex to its preferred visual field coordinates, as measured by the pRF center from the dataset averaged across attention conditions. Next, for a given focal attention condition, at each vertex we calculated the change in BOLD amplitude from distributed to focal attention conditions estimated by the GLM, averaged across the 49 mapping stimuli, and plotted this average value at the pRF location using a colormap. This produces a map of the visual field with, typically, several hundred points (each point is a surface vertex plotted at its pRF center). These data points were then resampled to a square grid using linear interpolation. Finally, to increase SNR, we averaged across these 2D visual field plots across the four attention conditions by rotating each one to align so that the attention direction was upward (90°).

#### Von Mises fitting procedure.

To quantify the 2D visualizations of attentional activity, we reduced the images to one spatial dimension, polar angle, constructing attentional tuning curves as a function of polar angle. For these analyses, we included the vertices whose preferred eccentricity was between 4° and 9° (close to the 6° eccentricity of the Gabor targets). For each of the target locations, we binned vertices by their polar angle distance from the target in 20° steps from −180° to 180°. Within each bin, we computed the attentional modulation as focal minus distributed response, averaging across the 49 mapping stimuli per vertex, and across vertices within the bin. The bin centers were computed as the average polar angle distance between the target location and the vertices’ pRF centers. These calculations produce one attentional tuning curve per target location for each visual area and each participant. Because the tuning curves did not differ systematically across the target locations, we averaged the four tuning curves per visual field map, and fit the averaged tuning curve with a difference of two von Mises functions, using the following equation using MATLAB’s *besseli* function:

(Eq 3) $$\hat{\text{y}}=\left[V_{\mu ,\kappa_{1}}(x)-V_{\mu ,\kappa_{2}}(x)\right]+B,\;\text{V}(\text{x};\mu ,\kappa )=S*\frac{\text{exp}(\kappa *\text{cos}(x-\mu ))}{2\pi *\text{besseli}(0,\kappa )}$$

where A is a scalar, B is an offset, is the Von Mises center (assumed to be the same for the positive and negative von Mises), and 1 and 2 are the concentrations of the two von Mises functions. These five parameters were fit for each visual field map in each participant by nonlinear least squares using MATLAB’s *fit* function. We defined the spread of attention in degrees of visual angle as the distance at which the difference of von Mises functions intercept the x-axis, meaning the transition point from attentional enhancement (focal > distributed) to attentional suppression (distributed > focal). To define the x-intercepts on either side of the center, we solved for f(x) = 0 twice, at [−60, 0] and [0, 60] lower and upper limits for the solution space. Lastly, we calculated the distance of two x-intercepts from each other to quantify the width along the x-axis. The group level estimates were obtained by averaging the estimated values across participants.

#### Definition of cortical target ROIs.

To quantify the retinotopic effects of attention, we created wedge-ROIs on the cortical surface by extracting the portions of cortex that represent four target locations. We used the averaged pRF solutions to extract a preferred visual field position for each vertex. In V1, V2, V3, hV4, V3A/B and LO1, we extracted all vertex pRF with a preferred eccentricity between 4° and 8°, and an angular preference of ±30° centered on each of the cardinal meridians (*UVM:* 60° – 120°, *LVM:* 240° – 300°, *LHM:* 150° – 210°, *RHM:* 330° – 360° & 0° – 30°).

#### Calculating the spatial shift effect.

We tested whether pRFs shifted towards an attentional target compared to their preferred position under distributed attention. For each vertex assigned to a visual map within a participant, we computed the changes in the best fitting pRF center across attention conditions. First, for each vertex, we extracted the estimated pRF center in *x* and *y* coordinates in degrees of visual angle, and the variance explained (*R2*) of each pRF model estimated for four focal and the distributed attention conditions. We only included vertices which met two criteria in all five pRF models: eccentricity in the range of 0.5° to 5.5° and variance explained greater than 0.25. Next, for each vertex and each of the four targets, we subtracted the distance between the distributed pRF center and target from the distance between focal pRF center and target. A negative value indicates that the pRF center got closer to a target when the target was attended. Last, we averaged the change in distance across vertices within an ROI and across attentional conditions. This analysis produces a summary metric of spatial shift per ROI.

#### Directional vector graphs of spatial shifts.

To investigate the pattern of spatial shifts within an ROI, we implemented a method from previous research^17^, by plotting the direction the average pRF centers within bins for two opposite focal attention conditions. For pairs of opposite focal conditions, we computed the average pRF position for each vertex (average of attend up/attend down or attend left/attend right). We then binned the vertex pRFs based on their averaged eccentricity (from 0.5° to 6.25° in steps of 1°) and polar angle (from 0° to 360° in steps of 45°) estimates, and averaged the center position across each bin separately for the two targets. We then plotted these two points for each bin connected by a line, and color coded the line by the congruence of the pRF center shift direction based on the changes in *x* coordinates for the horizontal targets, and in *y* coordinates for the vertical targets. If the pRF center shifts toward the attentional target, the attend left condition should have an *x* value that is smaller (or more negative) than the attend right condition (and vice versa for the attend right condition); and attend down condition should result in a smaller (or more negative) *y* than attend up condition (and vice versa for the attend up condition).

## Supplementary Material

Supplement 1

## Figures and Tables

**Table 1.** Estimated peak, trough and width of attentional spread within each visual field map.

ROI	Peak (%BOLD change)		Trough (%BOLD change)		Width (°)	
	Mean	68% Cl	Mean	68% CI	Mean	68% CI
V1	0.07	0.06, 0.09	−0.02	−0.04, −0.01	105	90, 120
V2	0.08	0.07, 0.09	−0.03	−0.04, −0.02	82	71, 92
V3	0.07	0.05, 0.08	−0.06	−0.09, −0.04	95	83, 107
hV4	0.04	0.03, 0.05	−0.12	−0.13, −0.09	74	64, 84
V3AB	0.06	0.04, 0.08	−0.09	−0.11,−0.09	89	75, 102
LO1	0.08	0.06, 0.1	−0.04	−0.05, −0.02	140	132, 142

## Data Availability

Code used to model, analyze, and visualize this data resides in https://github.com/ekintuncok/Attention_pRF. Raw and processed data reside in https://osf.io/g8b9v/.

## Footnotes

a We made measurements in V1, V2, V3, hV4, V3A/B, and LO1. V3A and V3B are considered to be distinct maps, but their boundary is not straightforward to identify. Hence for analysis purposes we group them as “V3A/B”; we analyze data from seven maps, grouped as six regions (maps) of interest.

## References

[1] Desimone R., and Duncan J. (1995). Neural mechanisms of selective visual attention. Annual Review of Neuroscience 18, 193–222. 10.1146/annurev.ne.18.030195.001205. 7605061

[2] Lennie P. (2003). The cost of cortical computation. Current Biology, 6, 493–497. 10.1016/s0960-9822(03)00135-0. 12646132

[3] Carrasco M. (2011). Visual attention: The past 25 years. Vision Research, 13, 1484–1525. 10.1016/j.visres.2011.04.012. PMC3390154 21549742

[4] Anton-Erxleben K., and Carrasco M. (2013). Attentional enhancement of spatial resolution: linking behavioural and neurophysiological evidence. Nat Rev Neurosci. 14(3), 188–200. 10.1038/nrn3443. 23422910 PMC3977878

[5] Dosher B.A., and Lu Z. L. (2000). Noise exclusion in spatial attention. Psychological Science, 11(2), 139–46. 10.1111/1467-9280.00229. 11273421

[6] Pestilli F., and Carrasco M. (2005). Attention enhances contrast sensitivity at cued and impairs it at uncued locations. Vision Research, 45(14), 1867. 10.1016/j.visres.2005.01.019. 15797776

[7] Ling S., Jehee J.F.M., and Pestilli F. (2014). A review of the mechanisms by which attentional feedback shapes visual selectivity. Brain Struct Funct, 220(3), 1237–1250. 10.1007/s00429-014-0818-5. 24990408 PMC4282837

[8] Maunsell J. (2015). Neuronal mechanisms of visual attention. Annu Rev Vis Sci, 1(1), 373–391. 10.1146/annurev-vision-082114-035431. 28532368 PMC8279254

[9] Kastner S., de Weerd P., Desimone R., and Ungerleider L.G. (1998). Mechanisms of directed dttention in the human extrastriate cortex as revealed by functional MRI. Science, 282, 108–111. 10.1126/science.282.5386.108. 9756472

[10] Reynolds J.H., Chelazzi L., and Desimone R. (1999). Competitive mechanisms subserve attention in macaque areas V2 and V4. Journal of Neuroscience, 19(5), 1736–1753. 10.1523/JNEUROSCI.19-05-01736.1999. 10024360 PMC6782185

[11] Tootel R.B.H., Hadjikhani N., Hall K.E., Marrett S., Vanduffel W., Vaughan T.J., and Dale A. M. (1998). The retinotopy of visual spatial attention, Neuron, 21(6), 1409–22. 10.1016/s0896-6273(00)80659-5. 9883733

[12] Bloem I.M., and Ling S. (2019). Normalization governs attentional modulation within human visual cortex. Nature Communications, 10(1), 5660. 10.1038/s41467-019-13597-1. PMC6906520 31827078

[13] Dugué L., Merriam E., Heeger D., and Carrasco M. (2020). Differential impact of endogenous and exogenous attention on activity in human visual cortex. Sci Rep, 10(1), 21274. 10.1038/s41598-020-78172-x. 33277552 PMC7718281

[14] Womelsdorf T., Anton-Erxleben K., Pieper F., and Treue S. (2006). Dynamic shifts of visual receptive fields in cortical area MT by spatial attention. Nature Neuroscience, 9(9), 1156–60. 10.1038/nn1748. 16906153

[15] Womelsdorf T., Anton-Erxleben K., and Treue S. (2008). Receptive field shift and shrinkage in macaque middle temporal area through attentional gain modulation. Journal of Neuroscience, 28(36), 8934–44. 10.1523/JNEUROSCI.4030-07.2008. 18768687 PMC6670861

[16] Sprague T.C., and Serences J.T. (2013). Attention modulates spatial priority maps in the human occipital, parietal and frontal cortices. Nat Neurosci. 16(12), 1879–87. 10.1038/nn.3574. 24212672 PMC3977704

[17] Klein B.P., Harvey B., and Dumoulin S. (2014). Attraction of position preference by spatial attention throughout human visual cortex. Neuron, 84(1), 227–237. 10.1016/j.neuron.2014.08.047. 25242220

[18] Kay K.N., Weiner K.S., and Grill-Spector K. (2015). Attention reduces spatial uncertainty in human ventral temporal cortex. Current Biology, 25(5), 595–600. 10.1016/j.cub.2014.12.050. 25702580 PMC4348205

[19] Vo V.A., Sprague T. C., and Serences J. T. (2017). Spatial tuning shifts increase the discriminability and fidelity of population codes in visual cortex. Journal of Neuroscience, 37(12), 3386–3401. 10.1523/JNEUROSCI.3484-16.2017. 28242794 PMC5373124

[20] Klein B.P., Fracasso A., Van Dijk J.A., Paffen C.L.E., Te Pas S.F. and Dumoulin S.O. (2018). Cortical depth dependent population receptive field attraction by spatial attention in human V1. NeuroImage, 176, 301–312. 10.1016/j.neuroimage.2018.04.055. 29709626

[21] Kastner S., Pinsk M.A., de Weerd P., Desimone R., and Ungerleider L.G. (1999). Increased activity in human visual cortex during directed attention in the absence of visual stimulation. Neuron, 22(4), 751–61. 10.1016/s0896-6273(00)80734-5. 10230795

[22] Serences J. T., Yantis S., Culberson A., and Awh E. (2004). Preparatory activity in visual cortex indexes distractor suppression during covert spatial orienting. Journal of Neurophysiology, 92(6), 3538–45. 10.1152/jn.00435.2004. 15254075

[23] Silver M.A., Ress D., and Heeger D.J. (2006). Neural correlates of sustained spatial attention in human early visual cortex. Journal of Neurophysiology, 97(1), 229–237. 10.1152/jn.00677.2006. 16971677 PMC1868502

[24] Capotosto P., Babiloni C., Romani G.L., and Corbetta M. (2009). Frontoparietal cortex controls spatial attention through modulation of anticipatory alpha rhythms. Journal of Neuroscience, 29(18), 5863–5872. 10.1523/JNEUROSCI.0539-09.2009. 19420253 PMC2692025

[25] Worden MS, Foxe JJ, Wang N, and Simpson GV. Anticipatory biasing of visuospatial attention indexed by retinotopically specific a-band electroencephalography increases over occipital cortex. Journal of Neuroscience, 95, 3844–3851. 10.1523/JNEUROSCI.20-06-j0002.2000. PMC6772495 10704517

[26] Gould I.C., Rushworth M.F., and Nobre A.C. (2011). Indexing the graded allocation of visuospatial attention using anticipatory alpha oscillations. Journal of Neurophysiology, 105(3), 1318–26. 10.1152/jn.00653.2010. 21228304 PMC3074422

[27] Zumer J.M., Scheeringa R., Schoffelen J.M., Norris D.G., and Jensen O. (2014). Occipital alpha activity during stimulus processing gates the information flow to object-selective cortex. PLoS Biology, 12(10), e1001965. 10.1371/journal.pbio.1001965. 25333286 PMC4205112

[28] Ress D, Backus B.T, and Heeger D.J. (2000). Activity in primary visual cortex predicts performance in a visual detection task. Nature Neuroscience, 3, 940–5. 10.1038/78856. 10966626

[29] Sapir A, Giovanni A., McAvoy M., Shulman G.L., and Corbetta M. (2005). Brain signals for spatial attention predict performance in a motion discrimination task. Proceedings of the National Academy of Sciences, 102(49), 17810–5. 10.1073/pnas.0504678102. PMC1308888 16306268

[30] Giesbrecht B., Weissman D.H., Woldorff M.G., and Mangun G.R. (2006). Pre-target activity in visual cortex predicts behavioral performance on spatial and feature attention tasks. Brain Research, 1080(1), 63–72. 10.1016/j.brainres.2005.09.068. 16412994

[31] Stokes M., Thompson R., Nobre A.C., and Duncan J. (2009). Shape-specific preparatory activity mediates attention to targets in human visual cortex. Proceedings of the National Academy of Sciences, 106(46), 19569–74. 10.1073/pnas.0905306106. PMC2772815 19887644

[32] Fannon S.P., Saron C.D., and Mangun G.R. (2008). Baseline shifts do not predict attentional modulation of target processing during feature-based visual attention. Front Hum Neurosci, 1, 7. 10.3389/neuro.09.007.2007. 18958221 PMC2525984

[33] Fox K.J., Birman D., and Gardner J.L. (2023). Gain, not concomitant changes in spatial receptive field properties, improves task performance in a neural network attention model. eLife, 12:e78392. 10.7554/eLife.78392. 37184221 PMC10241518

[34] Snyder A.C., Yu B.M., and Smith M.A. (2018). Distinct population codes for attention in the absence and presence of visual stimulation. Nature Communications, 9(1), 4382. 10.1038/s41467-018-06754-5. PMC6197235 30348942

[35] Carrasco M., Talgar C.P., and Cameron E.L. (2001). Characterizing visual performance fields: effects of transient covert attention, spatial frequency, eccentricity, task and set size. Spatial Vision, 15(1), 61–75. 10.1163/15685680152692015. 11893125 PMC4332623

[36] Himmelberg M.M., Winawer J., and Carrasco M. (2020). Stimulus-dependent contrast sensitivity asymmetries around the visual field. Journal of Vision, 20(9), 1–19. 10.1167/jov.20.9.18. PMC7533736 32986805

[37] Barbot A., Xue S., and Carrasco M. (2021). Asymmetries in visual acuity around the visual field. Journal of Vision, 21(1):2, 1–23. 10.1167/jov.21.1.2. PMC7794272 33393963

[38] Lee S., Blake R., and Heeger D.J. (2004). Traveling waves of activity in primary visual cortex during binocular rivalry. Nature Neuroscience, 8(1), 22–23. 10.1038/nn1365. 15580269 PMC1215464

[39] Stoll S., Infanti E., De Haas B., and Schwarzkopf D.S. (2022). Pitfalls in post hoc analyses of population receptive field data. NeuroImage, 8, 263, 119557. 10.1016/j.neuroimage.2022.119557. PMC7617406 35970472

[40] Lerma-Usabiaga G., Winawer J., and Wandell B.A. (2021). Population receptive field shapes in early visual cortex are nearly circular. Journal of Neuroscience, 41(11), 2420–2427. 10.1523/JNEUROSCI.3052-20.2021. 33531414 PMC7984596

[41] Posner M.I. (1980). Orienting of attention. The Quarterly Journal of Experimental Psychology, 32(1), 3–25. 10.1080/00335558008248231. 7367577

[42] Pestilli F., Ling S., and Carrasco M. (2009). A population-coding model of attention’s influence on contrast response: Estimating neural effects from psychophysical data. Vision Research, 49(10), 1144–1153. 10.1016/j.visres.2008.09.018. 18926845 PMC2743869

[43] Herrmann K., Montaser-Kouhsari L., Carrasco M., and Heeger D.J. (2010). When size matters: attention affects performance by contrast or response gain. Nature Neuroscience, 13(12), 1554–1559. 10.1038/nn.2669. 21057509 PMC3058765

[44] Fernández A., Hanning N.M., and Carrasco M. (2023). Transcranial magnetic stimulation to frontal but not occipital cortex disrupts endogenous attention. Proceedings of the National Academy of Sciences, 120(10), e2219635120. 10.1073/pnas.2219635120. PMC10013745 36853947

[45] Reynolds J.H., and Heeger. D.J. (2009). The normalization model of attention. Neuron, 61(2), 168–185. 10.1016/j.neuron.2009.01.002. 19186161 PMC2752446

[46] Itthipuripat S., Sprague T.C., and Serences J.T. (2019). Functional MRI and EEG index complementary attentional modulations. Journal of Neuroscience, 39(31), 6162–6179. 10.1523/JNEUROSCI.2519-18.2019. 31127004 PMC6668200

[47] Zanto T.P., Chadick J.Z., and Gazzaley A. (2013). Anticipatory alpha phase influences visual working memory performance. NeuroImage, 85, 794–802. 10.1016/j.neuroimage.2013.07.048. 23891902 PMC3859772

[48] Kok P., Jehee J.F.M., and de Lange F.P. (2012). Less is more: Expectation sharpens representations in the primary visual cortex. Neuron, 75(2), 265–270. 10.1016/j.neuron.2012.04.034. 22841311

[49] Kok P., Mostert P., and de Lange F.P. (2017). Prior expectations induce prestimulus sensory templates. Proceedings of the National Academy of Sciences, 114(39), 10473–10478. 10.1016/j.neuron.2012.04.034. PMC5625909 28900010

[50] Summerfield C., and Egner T. (2009). Expectation (and attention) in visual cognition. Trends in Cognitive Sciences, 13(9), 403–9. 10.1016/j.tics.2009.06.003. 19716752

[51] Bubic A., von Cramon Y.D., and Schubotz R.I. (2010). Prediction, cognition and the brain. Front. Hum. Neurosci, 4, 25. 10.3389/fnhum.2010.00025. 20631856 PMC2904053

[52] Williford T., and Maunsell J.H.R. (2006). Effects of spatial attention on contrast response functions in macaque area V4. Journal of Neurophysiology, 96(1), 40–54. 10.1152/jn.01207.2005. 16772516

[53] Thiele A., Pooresmaeili A., Delicato L.S., Herrero J.H., and Roelfsema P.R. (2009). Additive effects of attention and stimulus contrast in primary visual cortex. Cerebral Cortex, 19(12), 2970–81. 10.1093/cercor/bhp070. 19372142 PMC2774399

[54] McAdams C.J., and Maunsell J.H.R. (1999). Effects of attention on orientation-tuning functions of single neurons in macaque cortical area V4. Journal of Neuroscience, 19(1), 431–41. 10.1523/JNEUROSCI.19-01-00431.1999. 9870971 PMC6782389

[55] Treue S, and Martínez-Trujillo J.C. (1999). Feature-based attention influences motion processing gain in macaque visual cortex. Nature, 399(6736), 575–579. 10.1038/21176. 10376597

[56] Rabinowitz N.C., Goris R.L., Cohen M., and Simoncelli E.P. (2015). Attention stabilizes the shared gain of V4 populations. eLife, 4:e08998. 10.7554/eLife.08998. 26523390 PMC4758958

[57] Ruff D.A., and Cohen M.R. (2014). Attention can either increase or decrease spike count correlations in visual cortex. Nature Neuroscience, 17(11), 1591–1597. 10.1038/nn.3835. 25306550 PMC4446056

[58] Buracas G.T. and Boynton G.M. (2007). The effect of spatial attention on contrast response functions in human visual cortex. Journal of Neuroscience, 27(1), 93–97. 10.1523/JNEUROSCI.3162-06.2007. 17202476 PMC6672290

[59] Li X., Lu Z., Tjan B.S., Dosher B.A., and Chu W. (2008). Blood oxygenation level-dependent contrast response functions identify mechanisms of covert attention in early visual areas. Proceedings of the National Academy of Sciences, 105(16), 6202–7. 10.1073/pnas.0801390105. PMC2329700 18413602

[60] Murray S.O. (2008). The effects of spatial attention in early human visual cortex are stimulus independent. Journal of Vision, 8(10):2, 1–11. 10.1167/8.10.2. 19146344

[61] Pestilli F., Carrasco M., Heeger D.J., and Gardner J.L. (2011). Attentional enhancement via selection and pooling of early sensory responses in human visual cortex. Neuron, 72(5), 832–46. 10.1016/j.neuron.2011.09.025. 22153378 PMC3264681

[62] Itthipuripat S, Ester E.F., Deering S., and Serences J.T. (2014). Sensory gain outperforms efficient readout mechanisms in predicting attention-related improvements in behavior. Journal of Neuroscience, 34(40), 13384–98. 10.1523/JNEUROSCI.2277-14.2014. 25274817 PMC4180474

[63] Foster J.J., Thyer W., Wennberg J.A., and Awh E. (2021). Covert attention increases the gain of stimulus-evoked population codes. Journal of Neuroscience, 41(8), 1802–15. 10.1523/JNEUROSCI.2186-20.2020. 33441434 PMC8115885

[64] Hara Y., Pestilli F., and Gardner J.L. (2014). Differing effects of attention in single-units and populations are well predicted by heterogeneous tuning and the normalization model of attention. Front. Comput. Neurosci, 8, 12. 10.3389/fncom.2014.00012. 24600380 PMC3928538

[65] Foster J.J., and Ling S. (2022). Feature-based attention multiplicatively scales the fMRI-BOLD contrast-response function. Journal of Neuroscience, 42(36), 6894–906. 10.1523/JNEUROSCI.0513-22.2022. 35868860 PMC9464014

[66] Carrasco M, Penpeci-Talgar C., and Eckstein M. (2000). Spatial covert attention increases contrast sensitivity across the CSF: support for signal enhancement. Vision Research, 40(10–12), 1203–1215. 10.1016/s0042-6989(00)00024-9. 10788636 PMC3825514

[67] Suzuki M., and Gottlieb J. (2012). Distinct neural mechanisms of distractor suppression in the frontal and parietal lobe. Nature Neuroscience, 16(1), 98–104. 10.1038/nn.3282. 23242309 PMC4207121

[68] Noonan M.P., Adamian N., Pike A., Printzlau F., Crittenden B.M., Stokes M. (2016). Distinct mechanisms for distractor suppression and target facilitation. Journal of Neuroscience, 36(6), 1797–807. 10.1523/JNEUROSCI.2133-15.2016. 26865606 PMC4748069

[69] Schneider D., Herbst S.K., Klatt L.I., and Wöstmann M. (2021). Target enhancement or distractor suppression? Functionally distinct alpha oscillations form the basis of attention. Eur J of Neuroscience, 55(11–12), 3256–3265. 10.1111/ejn.15309. 33973310

[70] Himmelberg M.M., Winawer J., and Carrasco M. (2023). Polar angle asymmetries in visual perception and neural architecture. Trends in Neurosciences, 46(6), 445–458. 10.1016/j.tins.2023.03.006. 37031051 PMC10192146

[71] Kupers E.R., Carrasco M., and Winawer J. (2019). Modeling visual performance differences ‘around’ the visual field: A computational observer approach. PLoS Computational Biology, 15(5), e1007063. 10.1371/journal.pcbi.1007063. 31125331 PMC6553792

[72] Kupers E.R., Benson N.C., Carrasco M., and Winawer J. (2022). Asymmetries around the visual field: From retina to cortex to behavior. PLoS Computational Biology, 18(1), e1009771. 10.1371/journal.pcbi.1009771. 35007281 PMC8782511

[73] Liu T., Heeger D.J., and Carrasco M. (2006). Neural correlates of the visual vertical meridian asymmetry. Journal of Vision, 6(11), 1294–1306. 10.1167/5.1.1. 17209736 PMC1864963

[74] Benson N.C., Kupers E.R., Barbot A., Carrasco M., and Winawer. J. (2021). Cortical magnification in human visual cortex parallels task performance around the visual field. eLife, 10, e67685. 10.7554/eLife.67685. 34342581 PMC8378846

[75] Himmelberg M.M., Winawer J., and Carrasco M. (2022). Linking individual differences in human primary visual cortex to contrast sensitivity around the visual field. Nature Communications, 13(1), 3309. 10.1038/s41467-022-31041-9. PMC9192713 35697680

[76] Himmelberg M.M., Tünçok E., Gomez J., Grill-Spector K., Carrasco M., and Winawer J. (2023). Comparing retinotopic maps of children and adults reveals a late-stage change in how V1 samples the visual field. Nature Communications, 14(1), 1561. 10.1038/s41467-023-37280-8. PMC10030632 36944643

[77] Purokayastha S., Roberts M., and Carrasco M. (2021). Voluntary attention improves performance similarly around the visual field. Atten Percept Psychophys, 83(7), 2784–2794. 10.3758/s13414-021-02316-y. 34036535 PMC8514247

[78] Kleiner M., Brainard D., and Pelli D. (2007). What’s new in Psychtoolbox-3?.

[79] Kay K.N., Winawer J., Rokem A., Mezer A., and Wandell B.A. (2013). A two-stage cascade model of BOLD responses in human visual cortex. PLoS Comput Biol, 9(5), e1003079. 10.1371/journal.pcbi.1003079. 23737741 PMC3667759

[80] Kay K.N., Winawer J., Mezer A., and Wandell B.A. (2013). Compressive spatial summation in human visual cortex. Journal of Neurophysiology, 110(2), 481–494. 10.1152/jn.00105.2013. 23615546 PMC3727075

[81] Dumoulin S.O., and Wandell B.A. (2007). Population receptive field estimates in human visual cortex. NeuroImage, 39(2), 647–60. 10.1016/j.neuroimage.2007.09.034. 17977024 PMC3073038

[82] Prins N., and Kingdom F.A.A. (2018). Applying the model-comparison approach to test specific research hypotheses in psychophysical research using the Palamedes Toolbox. Frontiers in Psychology, 9, 1250. 10.3389/fpsyg.2018.01250. 30083122 PMC6064978

[83] Feinberg D.A., Moeller S., Smith S.M., Auerbach E., Ramanna S., Glasser M.F., Miller K.L., Ugurbil K., and Yacoub E. (2010). Multiplexed echo planar imaging for sub-second whole brain fMRI and fast diffusion imaging. PLoS ONE, 6(9), e15710. 10.1371/journal.pone.0015710. PMC3004955 21187930

[84] Moeller S., Yacoub E., Olman C.A., Auerbach E., Strupp J., Harel N., and Ugurbil K. (2010). Multiband multislice GE-EPI at 7 tesla, with 16-fold acceleration using partial parallel imaging with application to high spatial and temporal whole-brain fMRI. Magnetic Resonance in Medicine, 63(5), 1144–1153. 10.1002/mrm.22361. 20432285 PMC2906244

[85] Xu J., Moeller S., Auerbach E.J., Strupp J., Smith S.M., Feinberg D.A., Yacoub E., and Ugurbil K. (2013). Evaluation of slice accelerations using multiband echo planar imaging at 3T. NeuroImage, 83, 991–1001. 10.1016/j.neuroimage.2013.07.055. 23899722 PMC3815955

[86] Gorgolewski K.J., Auer T., Calhoun V.D., Craddock R.C., Das S., Duff E.P., … Poldrack R. A. (2016). The brain imaging data structure, a format for organizing and describing outputs of neuroimaging experiments. Sci Data, 3(1), 160044. 10.1038/sdata.2016.44. 27326542 PMC4978148

[87] Esteban O., Markiewicz C.J., Blair R.W., Moodie C.A., Isik A.I., Erramuzpe A., … Gorgolewski K. J. (2018). fMRIPrep: a robust preprocessing pipeline for functional MRI. Nature Methods, 16(1), 111–116. 10.1038/s41592-018-0235-4. 30532080 PMC6319393

[88] Dale A.M., Fischl B., and Sereno M.I. (1999). Cortical surface-based analysis: I. Segmentation and surface reconstruction. NeuroImage, 9(2), 179–194. 10.1006/nimg.1998.0395. 9931268

[89] Greve D.N., and Fischl B. (2009). Accurate and robust brain image alignment using boundary-based registration. NeuroImage, 48(1), 63–72. 10.1016/j.neuroimage.2009.06.060. 19573611 PMC2733527

[90] Cox R.W., and Hyde J.S. (1997). Software tools for analysis and visualization of fMRI data. NMR Biomed, 10(4–5), 171–8. 10.1002/(sici)1099-1492(199706/08)10:4/5<171::aidnbm453>3.0.co;2-l. 9430344

[91] Recht S., Mamassian P., and de Gardelle V. (2019). Temporal attention causes systematic biases in visual confidence. Sci Rep, 9(1), 11622. 10.1038/s41598-019-48063-x. 31406265 PMC6690997

[92] Hautus M.J. (1995). Corrections for extreme proportions and their biasing effects on estimated values of d′. Behavior Research Methods, Instruments, & Computers, 27(1), 46–51. 10.3758/BF03203619.

[93] Brown G.S., and White K.G. (2005). The optimal correction for estimating extreme discriminability. Behavior Research Methods, 37(3), 436–49. 10.3758/BF03192712. 16405138

[94] Kay K.N., Rokem A., Winawer J., Dougherty R.F., and Wandell B.A. (2013). GLMdenoise: a fast, automated technique for denoising task-based fMRI data. Frontiers in Neuroscience, 7, 247. 10.3389/fnins.2013.00247. 24381539 PMC3865440

[95] Parent R. (2012). Computer Animation: Algorithms and Techniques (Morgan Kauffman)

[96] Benson N.C., and Winawer J. (2018). Bayesian analysis of retinotopic maps. eLife, 7, e40224. 10.7554/eLife.40224. 30520736 PMC6340702

[97] Benson N.C., Yoon J.M.D., Forenzo D., Engel S.A., Kay K.N., and Winawer J. (2022). Variability of the surface area of the V1, V2, and V3 maps in a large sample of human observers. Journal of Neuroscience, 42(46), 8629–8646. 10.1523/JNEUROSCI.0690-21.2022. 36180226 PMC9671582

[98] Winawer J., and Witthoft N. (2015). Human V4 and ventral occipital retinotopic maps. Vis Neurosci, 32, E020. 10.1017/S0952523815000176. 26241699 PMC4730874

[99] Larsson J., and Heeger D.J. (2006). Two retinotopic visual areas in human lateral occipital cortex. Journal of Neuroscience, 26(51), 13128–13142. 10.1523/JNEUROSCI.1657-06.2006. 17182764 PMC1904390

[100] Efron B. (1981). Non parametric estimates of standard Error: The jackknife, the bootstrap and other methods. Biometrika, 68(3), 589–599. 10.2307/2335441.

[101] Sit Y.F., Chen Y., Geisler W.S., Miikkulainen R., and Seidemann E. (2009). Complex dynamics of V1 population responses explained by a simple gain-control model. Neuron, 64(6), 778–80. 20064399 10.1016/j.neuron.2009.08.041 PMC2807412

