# Supplementary material for: Spatial attention alters visual cortical representation during target anticipation
Source: bioRxiv. 2024 Mar 7:2024.03.02.583127. Preprint. [Version 2] doi: 10.1101/2024.03.02.583127 (PMC10942396; doi:10.1101/2024.03.02.583127)
Supplement: Supplementary file 1 [file media-1.pdf]

Supplementary Materials

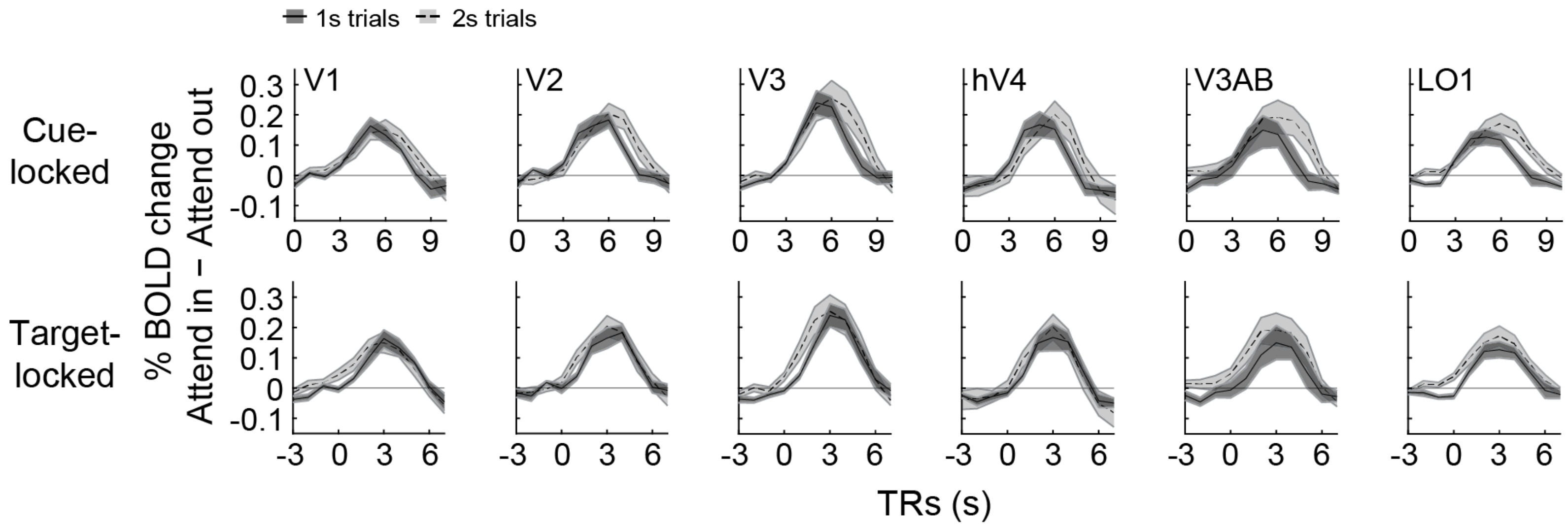

Fig 1. Attentional modulation of BOLD in 1-s and 2-s mapping stimulus bar trials in all visual field maps, cue-locked (top), and target-locked and shifted (bottom). In the original manuscript, we only plot the data from V3 in **Figure 4B.** Note that all visual field maps show a similar temporal pattern of attentional modulation. This figure is produced by *fig4_A_TTA_bar_duration.m*.

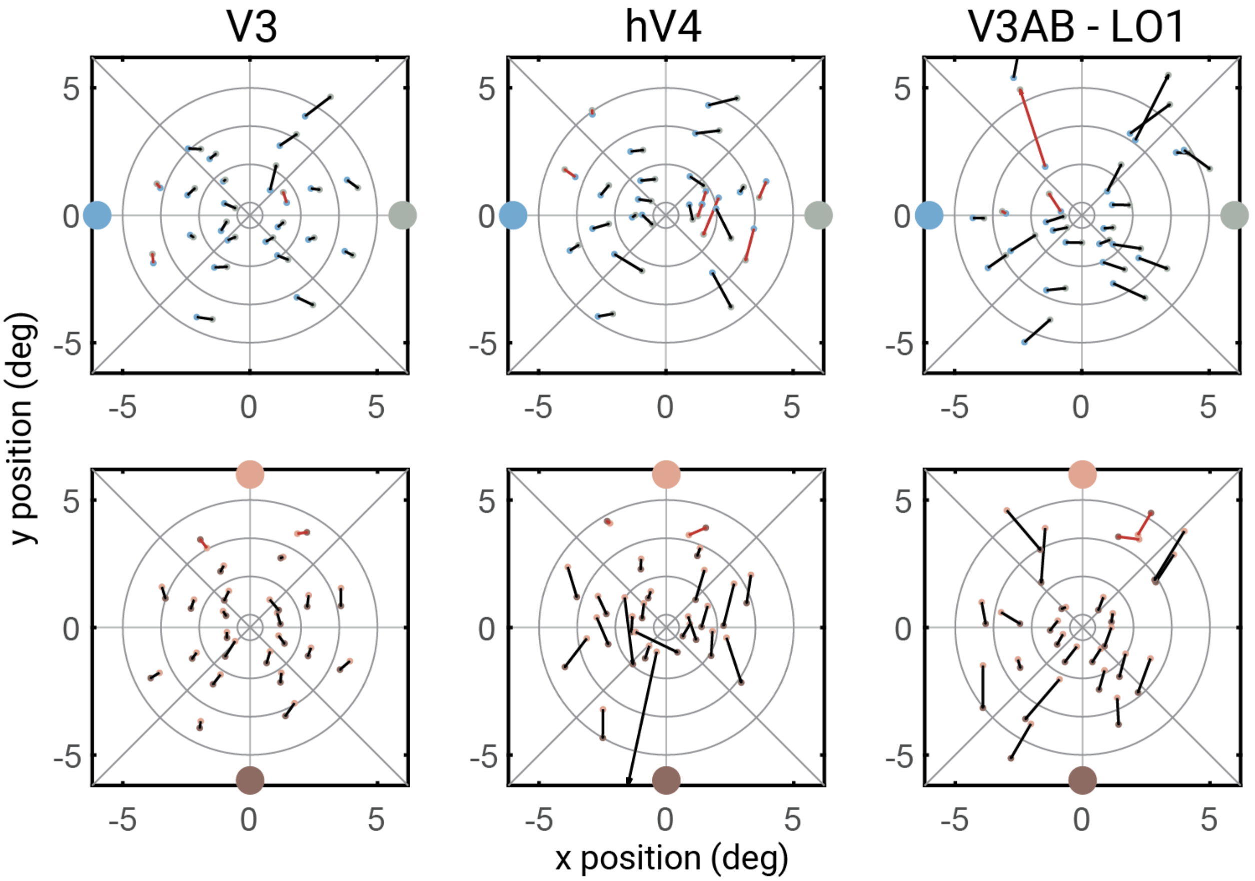

Fig 2. PRF center shifts visualized along the horizontal (top) and vertical (bottom) attentional shift axes in V3, hV4 and V3A/B/LO1 averaged. At each bin, the vector color indicates whether the center shift occurred towards (black lines) or away (red lines) from the attentional target. Here the binning of vertices is based on the pRF preferred center data estimated with distributed attention condition, which was estimated independently of the focal attention conditions. Estimating the bin centers with an independent condition circumvents potential circularity errors from analyzing binned data[1]. We reduced the number of eccentricity bins for this analysis because assigning vertices to bins based on an independent dataset naturally reduces the overlap between preferred center estimates resulting in more noise than the data plotted in **Figure 7B**. Overall, pRF center shifts are still in the expected direction, in line with the attentional shift. This figure is produced by *fig7_B_directional_vector_graphs_of_shifts.m* (input *distributed* when the binning method is prompted).

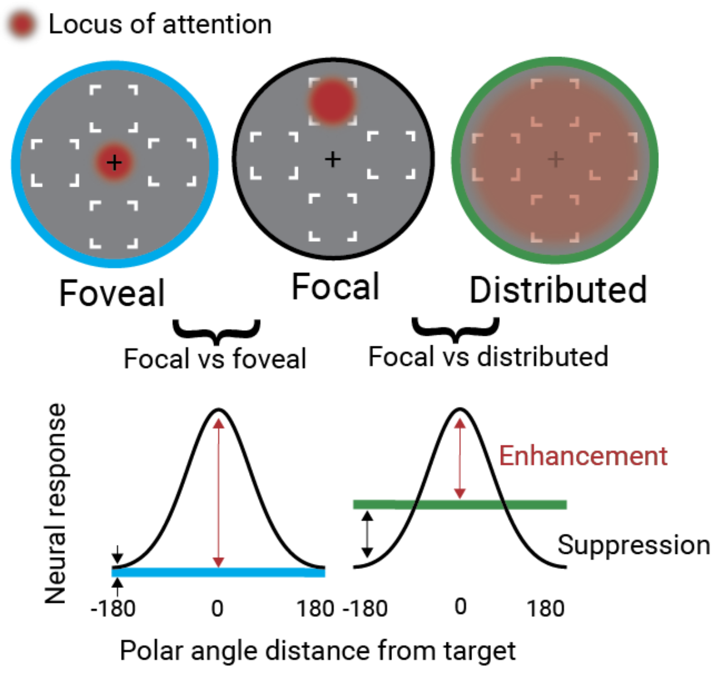

Fig 3. A schematic showing enhancement and suppression profiles of spatial attention relative to two different baseline conditions. The upper panel shows the attentional distribution in three conditions - foveal attention, focal attention to a peripheral location, and distributed attention. The lower panel shows hypothetical neural response patterns as a function of polar angle distance from a target in the upper target location, similar to our data plots in Figure 4. The black curve is the response when attending focally (to the upper target location), and hence peaks at 0 deg (target location). The blue line (left plot) shows a reduced neural response assuming that foveal attention has caused a withdrawal of peripheral responses. The green line (right plot) shows an elevated neural response at all locations. If foveal attention is the baseline, there will appear to be a large enhancement effect and no suppression. If the distributed condition is the baseline, there will be both an enhancement and a suppression. The distributed baseline better enables the measure of local distractor suppression.

ROI	R^2	Rising latency (2s – 1s) Cue-locked	Rising latency (2s – 1s) Target-locked	Falling latency (2s – 1s) Cue-locked	Falling latency (2s – 1s) Target-locked
V1	0.95	-1.05 [-2.11, 0.01]	-1.85 [-3.00, -0.66]	0.89 [0.57, 1.20]	-0.13 [-0.46, 0.19]
V2	0.96	0.73 [-0.04, 1.47]	-0.70 [-1.97, 0.45]	0.58 [0.46, 0.70]	-0.44 [-0.54, -0.33]
V3	0.97	-0.20 [-0.83, 0.36]	-1.14 [-1.76, -0.58]	0.98 [0.81, 1.14]	-0.02 [-0.19, 0.12]
hV4	0.96	-0.05 [-0.77, 0.70]	-1.26 [-2.15, -0.38]	0.71 [0.49, 0.88]	-0.33 [-0.52, -0.16]
V3AB	0.94	-0.92 [-2.71, 0.95]	-1.94 [-3.64, -0.38]	1.15 [0.92, 1.39]	0.15 [-0.07, 0.39]
LO1	0.98	-0.31 [-0.86, 0.16]	-1.45 [-1.98, -0.97]	0.78 [0.58, 0.96]	-0.22 [-0.40, -0.04]

Table 1. Estimated attentional rising and falling latency difference between 2-s and 1-s mapping stimulus trials for cue- and target-locked time series analysis within each visual field map. Attentional modulation starts rising around the same time for two different bar durations when it is cue-locked, and it starts falling around the same time for two bar durations when it is target-locked.

References

1. Stoll, S., Infanti, E., De Haas, B., & Schwarzkopf, D. S. (2022). Pitfalls in post hoc analyses of population receptive field data. *Neuroimage*, *263*, 119557.
